# Molecular characterization of systemic sclerosis esophageal pathology identifies inflammatory and proliferative signatures

**DOI:** 10.1186/s13075-015-0695-1

**Published:** 2015-07-29

**Authors:** Jaclyn N. Taroni, Viktor Martyanov, Chiang-Ching Huang, J. Matthew Mahoney, Ikuo Hirano, Brandon Shetuni, Guang-Yu Yang, Darren Brenner, Barbara Jung, Tammara A. Wood, Swati Bhattacharyya, Orit Almagor, Jungwha Lee, Arlene Sirajuddin, John Varga, Rowland W. Chang, Michael L. Whitfield, Monique Hinchcliff

**Affiliations:** Department of Genetics, Geisel School of Medicine at Dartmouth, 1 Rope Ferry Road, Hanover, NH 03755 USA; Zilber School of Public Health, University of Wisconsin, 1240 N 10th Street, Milwaukee, WI 53205 USA; Department of Neurological Sciences, College of Medicine, University of Vermont, 89 Beaumont Avenue, Burlington, VT 05405 USA; Department of Medicine, Division of Gastroenterology and Hepatology, Northwestern University Feinberg School of Medicine, 676 N. Saint Clair Street, Suite 1400, Chicago, IL 60611 USA; Department of Pathology, Northwestern University Feinberg School of Medicine, 303 E. Chicago Avenue, Ward- 3-140, Chicago, IL 60611 USA; Department of Medicine, Division of Gastroenterology, University of Illinois Chicago, 808 S Wood Street, Chicago, Illinois 60612 USA; Department of Medicine, Division of Rheumatology, Northwestern University Feinberg School of Medicine, 240 E. Huron Street, Suite M300, Chicago, IL 60611 USA; Department of Preventive Medicine, Northwestern University Feinberg School of Medicine, 680 N. Lake Shore Drive, Suite 1400, Chicago, IL 60611 USA; Institute for Public Health and Medicine, Northwestern University, 633 N. St. Clair Street, 18th floor, Chicago, IL 60611 USA; Department of Radiology, Northwestern University Feinberg School of Medicine, 676 N. St. Clair Street, Chicago, IL 60611 USA; Department of Physical Medicine and Rehabilitation, Northwestern University Feinberg School of Medicine, 710 N. Lake Shore Drive, Chicago, IL 60611 USA

## Abstract

**Introduction:**

Esophageal involvement in patients with systemic sclerosis (SSc) is common, but tissue-specific pathological mechanisms are poorly understood. There are no animal scleroderma esophagus models and esophageal smooth muscle cells dedifferentiate in culture prohibiting in vitro studies. Esophageal fibrosis is thought to disrupt smooth muscle function and lead to esophageal dilatation, but autopsy studies demonstrate esophageal smooth muscle atrophy and the absence of fibrosis in the majority of SSc cases. Herein, we perform a detailed characterization of SSc esophageal histopathology and molecular signatures at the level of gene expression.

**Methods:**

Esophageal biopsies were prospectively obtained during esophagogastroduodenoscopy in 16 consecutive SSc patients and 7 subjects without SSc. Upper and lower esophageal biopsies were evaluated for histopathology and gene expression.

**Results:**

Individual patient’s upper and lower esophageal biopsies showed nearly identical patterns of gene expression. Similar to skin, inflammatory and proliferative gene expression signatures were identified suggesting that molecular subsets are a universal feature of SSc end-target organ pathology. The inflammatory signature was present in biopsies without high numbers of infiltrating lymphocytes. Molecular classification of esophageal biopsies was independent of SSc skin subtype, serum autoantibodies and esophagitis.

**Conclusions:**

Proliferative and inflammatory molecular gene expression subsets in tissues from patients with SSc may be a conserved, reproducible component of SSc pathogenesis. The inflammatory signature is observed in biopsies that lack large inflammatory infiltrates suggesting that immune activation is a major driver of SSc esophageal pathogenesis.

**Electronic supplementary material:**

The online version of this article (doi:10.1186/s13075-015-0695-1) contains supplementary material, which is available to authorized users.

## Introduction

The esophagus is frequently affected in patients with systemic sclerosis (SSc; scleroderma), but the pathogenesis is poorly understood [1–3]. A scleroderma colonic fibrosis mouse model has been described, but no animal models of scleroderma esophageal disease have been developed [4]. Esophageal manometry reveals weak to absent peristaltic activity and loss of lower sphincter tone in SSc patients that predisposes to gastroesophageal reflux (GER) [1]. Proton pump inhibition (PPI) effectively treats GER, but has little effect on esophageal dysmotility [3]. There is an unmet need for biomarkers that predict development of SSc esophageal dysmotility, methods that will yield insights into pathogenesis, and novel strategies to prevent and treat SSc esophageal disease.

The replacement of smooth muscle with collagen in the esophageal mucosa (fibrosis) is thought to precipitate SSc esophageal dysmotility, but autopsy and functional studies demonstrate that smooth muscle atrophy is the predominant pathology [5–7]. Hypotheses for the development of smooth muscle atrophy include vasculopathy with resultant denervation, production of autoantibodies targeting smooth muscle and/or entrapment and destruction of smooth muscle by fibrosis [2].

Whole-genome gene expression profiling of skin biopsies in SSc has led to the identification of SSc ‘intrinsic subsets’ (fibroproliferative, inflammatory, limited and normal-like) that are distinct from clinically identified subtypes (limited cutaneous/lc versus diffuse cutaneous/dc) defined based upon skin involvement and serum autoantibodies [8]. Different molecular pathways underlie the inflammatory and fibroproliferative subsets [9, 10]. Specific gene expression signatures in skin have been shown to be associated with clinical improvement during mycophenolate mofetil (Cellcept™) and imatinib mesylate (Gleevec™) therapy [11, 12].

We hypothesized that histopathological and gene expression studies in esophageal biopsies from patients with SSc would provide insight into pathological processes and determine whether they are similar between skin and esophagus. Here, we present the first comprehensive analysis of histopathological and molecular changes in SSc-associated esophageal disease to our knowledge.

## Methods

The Northwestern Institutional Review Board approved the study and ensured compliance with the principles of the Declaration of Helsinki. Subjects gave written informed consent to undergo esophageal biopsies. Sixteen patients who met 2013 American College of Rheumatology criteria for SSc were studied [13]. Seven patients without SSc were enrolled as a comparator disease group. Subjects underwent esophagogastroduodenoscopy (EGD) with esophageal biopsies for a clinical indication (Additional file 1). Esophagitis was diagnosed during EGD for patients that met Los Angeles classification criteria [14]. For research purposes, one additional biopsy pair (upper and lower esophagus) was placed in RNAlater (Applied Biosystems, Ambion®, Carlsbad, CA, USA) and used for DNA microarray analysis; another biopsy pair was placed in formalin for histological analyses.

Age, sex, ethnicity, body mass index, smoking history, presence of GER symptoms, use of PPI, and gastrointestinal (GI) symptom duration (defined as interval between GI symptom onset and EGD) were abstracted from the electronic medical record. Modified Rodnan skin score (mRSS), SSc disease duration (defined as interval between first non-Raynaud symptom and EGD), SSc subset (lc or dc), and immune modulatory treatment including mycophenolate mofetil exposure (never, past or current) were abstracted for SSc patients. Serum antinuclear antibodies (ANA), anti-topoisomerase I, anticentromere, and anti-RNA polymerase III antibody titers were measured by indirect immunofluorescence at Specialty Laboratories (Valencia, CA, USA).

Pulmonary function tests (PFT) and lung high-resolution computed tomography (HRCT) examinations were obtained when clinically indicated. A chest radiologist who was blinded to clinical data determined the presence or absence of a patulous esophagus and interstitial lung disease (ILD) on HRCT examinations. A patulous esophagus was reported if the luminal diameter of the air or fluid-filled esophagus measured >10 mm in the coronal plane between the level of the aortic arch and the cardiac ventricles, >15 mm in the coronal plane between the level of the cardiac ventricles and the lower esophageal sphincter, or if an air-fluid level was present [15–17]. Pulmonary fibrosis was reported if there was ground-glass opacity or reticulation in nondependent portions of lung or if there was ground-glass opacity and reticulation in dependent portions of lung that persisted on prone imaging [18, 19]. The presence of honeycombing and traction bronchiectasis was consistent with fibrosis [18, 19].

### Esophageal biopsies

Esophageal biopsies were obtained using standard sized, Radial Jaw 4 biopsy forceps (Boston Scientific, Boston, MA, USA). Upper (within 10 cm of the esophageal inlet) and lower (5 cm proximal to the squamocolumnar junction) esophageal biopsies were obtained. Tissues were paraffin-embedded, and 4-μm sections were stained with hematoxylin and eosin (H&E). Photomicrographs of H&E-stained esophageal biopsies (20× and 40× magnification) were obtained using an Olympus BX45 microscope and Olympus DP70 camera (Olympus America, Inc., Center Valley, PA, USA).

In order to identify histological changes that may be SSc-specific and not attributable to esophagitis, three approaches were undertaken. First, the presence of a hiatal hernia and/or esophagitis on gross examination of the esophageal lumen at the time of EGD was considered evidence for esophagitis [14]. Second, a GI pathologist who was blinded to clinical data scored esophageal biopsies for degree of basal cell hyperplasia (0 = basal cells restricted to basal layer, 1 = basal cells above basal layer but penetrating <1/3 thickness of squamous epithelium, 2 = basal cells penetrating into 1/3–2/3 of thickness of squamous epithelium, 3 = basal cells infiltrating cells >2/3 into squamous epithelium). Third, the area with the greatest intraepithelial lymphocyte density on lower power (10× magnification) was identified, and the number of lymphocytes per high-power field (HPF) was counted [5, 20–23]. A finding of grade ≥1 basal cell hyperplasia or ≥10 lymphocytes/HPF was considered pathological evidence for esophagitis [5, 20–23]. A pathologist also assessed H&E-stained sections for yeast and pseudohyphae consistent with candida esophagitis. Esophageal biopsies were also scored for the degree of collagen deposition in the lamina propria (0 = no, 1 = mild, 2 = moderate and 3 = severe) to assess whether patients with SSc have more fibrosis than patients without SSc.

### Microarray processing and analysis

RNA was prepared from esophageal biopsies as previously reported for SSc skin biopsies [11]. A total of 200 ng total RNA was amplified and labeled using the Agilent Quick Amp Labeling Kit [8] and co-hybridized to Agilent Whole Human Genome (4 × 44 K) Microarrays (G4112F) (Agilent Technologies, Santa Clara, CA, USA) [11]. Data were log_2_ lowess normalized and filtered for probes with relative intensity greater than or equal to 1.5 of the median spot background in Cy3 or Cy5 channels. Data were multiplied by −1 to convert to log_2_(Cy3/Cy5) ratios. Probes with >20 % missing data were excluded.

Systematic biases resulting from technical artifacts were detected by multidimensional scaling analyses (MDS) in R [24]. Two biopsies (Eso05 lower and SSc12 upper) were identified as outliers by MDS and subsequently excluded from all analyses. Missing values in the remaining expression data were imputed via *k*-nearest neighbor algorithm using a GenePattern module with default parameters [25]. Batch effects (potential sources of nonbiological experimental variation) in the expression data were adjusted using ComBat run as a GenePattern module using nonparametric settings [26]. The statistical significance of batch bias before (*p* <0.001) and after (*p* = 0.997) adjustment with ComBat was assessed with guided principal component analysis (gPCA; Additional file 2) [27].

Transcripts that were differentially expressed between patients with and without SSc (unpaired *t* test) and transcripts differentially expressed between SSc patients’ upper and lower biopsies (paired *t* test) were identified using the GenePattern module Comparative Marker Selection using log-transformed data with all other settings set to default [28]. Uncorrected *p* values are reported in the results for the unpaired *t* test between SSc and controls. Hierarchical clustering was performed with Cluster 3.0 using uncentered Pearson correlation as the distance metric and average linkage [29]. Data were displayed with Java TreeView version 1.1.6r2 [30].

Statistical significance of clustering (SigClust) is designed to assess the significance of splitting a data set into two clusters. Cluster membership was assigned by running *k*-means, the basis of SigClust. The *p* values reported are the simulated SigClust *p* values based on Gaussian quantiles. Consensus clustering allows for the examination of the stability of clusters by identifying the ‘consensus’, or the agreement in cluster assignment between multiple runs of the algorithm in which the number of clusters, or *k*, is increased. Consensus clustering was performed on the intrinsic genes via the Consensus Clustering module (version 7) in GenePattern using the hierarchical clustering algorithm and Pearson distance [31]. Max *k* was set to 10 and all other settings were set to default.

Intrinsic gene selection was performed using a custom Matlab script [11]. SigClust and consensus clustering were used to determine the number of significant clusters within the cohort [8, 31, 32]. Significance analysis of microarrays (SAM) was run as an Excel plug-in with 300 permutations (multiclass response type) to identify genes significantly differentially expressed between subsets of SSc patients. Functional enrichment analysis of differentially expressed probes was performed with g:GOSt within g:Profiler [33]. Functional terms with *p* value <0.05 (corrected for multiple testing via default g:GOSt method) were considered.

### Statistical analyses

Categorical variables were compared by Fisher’s exact test due to the small sample size. Continuous variables were expressed as mean and standard deviation. Normality of continuous variables was assessed by Shapiro-Wilk test and data were considered non-normal when *p* <0.05. Statistically significant differences between patients with and without the inflammatory signature were assessed by *t* tests with Welch correction or Wilcoxon rank sum test. For all analyses, a two-sided *p* value <0.05 was considered significant. SAS version 9.3 (SAS Institute, Cary, NC, USA), R version 2.15.3, and GraphPad Prism 6.0 (GraphPad Software, San Diego, CA, USA) were used.

### Quantitative reverse transcriptase-polymerase chain reaction (qRT-PCR)

RNA was reverse-transcribed to cDNA [34] and amplicons were analyzed in duplicate by PCR using SYBR Green PCR Master Mix (Applied Biosystems, Foster City, CA, USA) on the Applied Biosystems 7500 Prism Sequence Detection System with primers as indicated in Table S2 (see Additional file 3). Results are fold change relative to the mean expression for upper and lower esophageal biopsies for subject ESO3 (Additional file 1). This subject with normocytic anemia was selected for the normalization procedure because she was not receiving PPI therapy, EGD revealed a grossly normal esophagus, and no histological evidence for esophagitis was present.

### Data availability

The expression data are available from NCBI Gene Expression Omnibus (GSE68698). This series reflects the most complete version of the dataset (46 arrays, as used in Additional file 4). Other expression data matrices used throughout the manuscript are included as part of Additional file 5.

## Results

### Subjects and clinical characteristics

Sixteen consecutively enrolled subjects with SSc and seven subjects without SSc undergoing EGD with esophageal biopsies for a clinical indication were studied. Two patients without SSc (Eso2 and Eso5) had rheumatic diseases (systemic lupus erythematosus and undifferentiated seronegative spondyloarthropathy. Clinical and demographic features are summarized in Additional file 1. Ninety-four percent of the patients with SSc and 71 % of patients without SSc were women. Mean GI symptom duration was 71 months for patients with SSc and 25 months for patients without SSc. Mean SSc disease duration at the time of esophageal biopsies was 106 months. Sixty-three percent of the SSc patients had dcSSc. All SSc patients had positive serum ANA. No subjects were current smokers. All SSc patients and 57 % of the patients without SSc were using PPIs at the time of biopsies (*p* = 0.02). Chest HRCT was performed within 1 year of esophageal biopsies in all SSc patients and one patient without SSc. Thirteen out of 16 (81 %) patients with SSc had a patulous esophagus.

### Molecular overview

To identify genes that are specific to SSc esophageal disease and avoid confounding with genes that are potentially related to autoimmunity, biopsies from two patients without SSc (Eso2 and Eso5) but with rheumatic diseases were excluded. Thus, gene expression in 43 esophageal biopsies from 16 patients with SSc and 5 patients without SSc was analyzed. Analysis of differential gene expression identified 1903 probes (1350 unique genes) significantly different between SSc and control biopsies (*p* <0.05, *t* test) Additional file 6. Genes upregulated in SSc biopsies included *IL27*, *IFNAR1*, and *PDGFRA*. Genes downregulated in SSc included *CCL2* and several human leukocyte antigen genes. This analysis was repeated including Eso2 and Eso5 biopsies, which resulted in fundamentally similar results that are included as (Additional file 4).

Given the overlapping pathology in SSc and non-SSc esophageal diseases, some shared gene expression was expected. Similar expression patterns were observed in patients with and without SSc (Additional file 6B), including genes involved in immune response such as *IRAK1, TAB1* and *RELA* (green bar) and *CD59, CCL4* and *THBS1* (red bar). Gene expression within SSc biopsies was heterogeneous resulting in three apparent groups of patients (Additional file 6). In order to identify the most robust gene expression signatures among SSc esophageal biopsies in the absence of potentially confounding and overlapping pathologies, we analyzed SSc samples alone.

### Molecular heterogeneity of SSc esophageal disease

To determine whether gene expression in upper and lower esophageal biopsies is similar in patients with SSc, we conducted paired analyses. Only the 15 patients with SSc that had both biopsies pass quality control filters were considered for the remainder of the analyses. Sixteen biopsy pairs were analyzed because one patient underwent biopsies at two time points. Similar to skin, gene expression in paired upper and lower esophageal biopsies from individuals was more similar than between individuals (Additional file 7). In fact, 15 out of 16 (94 %) paired upper and lower esophageal biopsies clustered together (Additional file 7A). As SSc patients typically have lower esophageal involvement, a paired *t* test was used to detect genes most differentially expressed between patients’ upper and lower biopsies. A total of 1479 probes with a false discovery rate (FDR) <5 % were selected and arrays were hierarchically clustered. Despite purposely selecting differentially expressed genes between upper and lower biopsies, 14 out of 16 (87.5 %) pairs clustered together (Additional file 8).

**Fig. 1:**
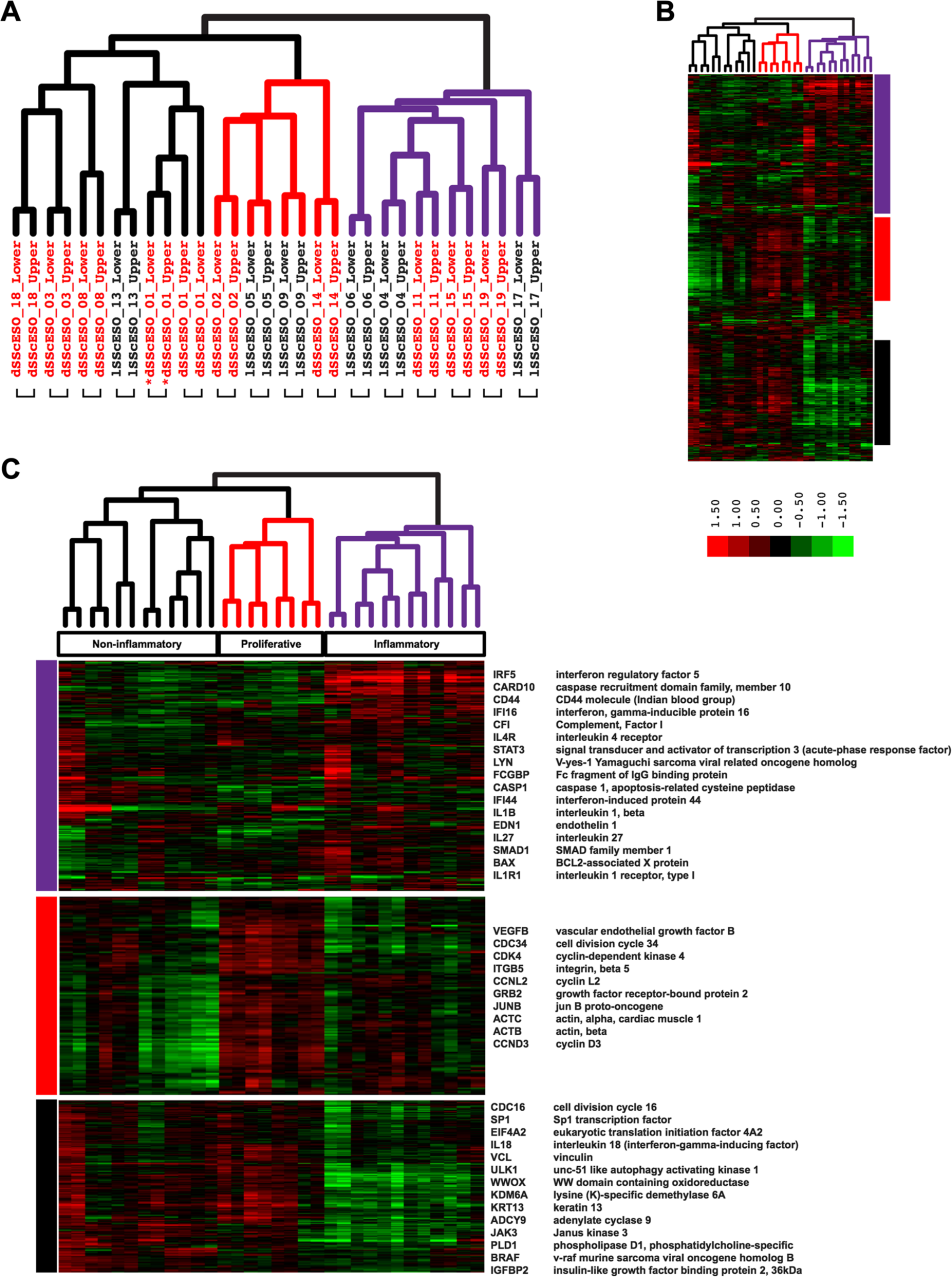
Esophageal intrinsic genes. A total of 2240 probes representing 2085 unique transcripts with the most similar expression between upper and lower biopsies for an individual but with the most dissimilar expression between individuals, termed ‘intrinsic’, were identified (false discovery rate (FDR) <1.1 %). An *asterisk* indicates samples obtained at 6 months. **a** Sample dendrogram, leaves are colored by group membership: *red* – samples from proliferative subset, *purple* – samples from inflammatory subset, *black* – samples from noninflammatory subset. Brackets indicate biopsies from the upper and lower esophagus for an individual that clustered together. **b** Overview of hierarchically clustered probes. **c** Selected gene clusters: *purple*, upregulated in inflammatory patients; *red*, upregulated in a proliferative subset of patients; *black*, downregulated in inflammatory patients. Additional file 9 contains the full list of transcripts

In order to quantify SSc esophageal heterogeneity, ‘intrinsic subset analysis’ of the data was performed [8]. Briefly, 2240 probes (2085 unique genes) with the most similar expression between upper and lower esophageal biopsies for an individual patient, but the most dissimilar expression between individuals, were identified (FDR <1.1 %). Using this approach, patients were clustered into three distinct groups based on the expression patterns of the intrinsic genes (Fig. 1).

An inflammatory group was comprised of patients 4, 6, 11, 15, 17, 19 (Fig. 1a and b). Genes with increased expression in this group included interferon-induced proteins (*IFI16* and *IFI44*), components of the inflammasome pathway (*CASP1* and *IL1B*), and other genes related to inflammation (Fig. 1c, and Additional file 9). The remaining patients formed two subgroups. Patients 2, 5, 9 and 14 formed a proliferative group, with increased expression of genes indicative of proliferating cells (*CDK4* and *CDC34*) as well as cyclins *CCND3* and *CCNL2* (Fig. 1c). Patients 1, 3, 8, 13 and 18 clustered into a noninflammatory group that showed high expression of genes indicative of cell growth (*BRAF*, *CDC16* and *SP1*) (Fig. 1c), suggesting a possible functional overlap with the proliferative group that displayed a similar expression pattern.

**Fig. 2:**
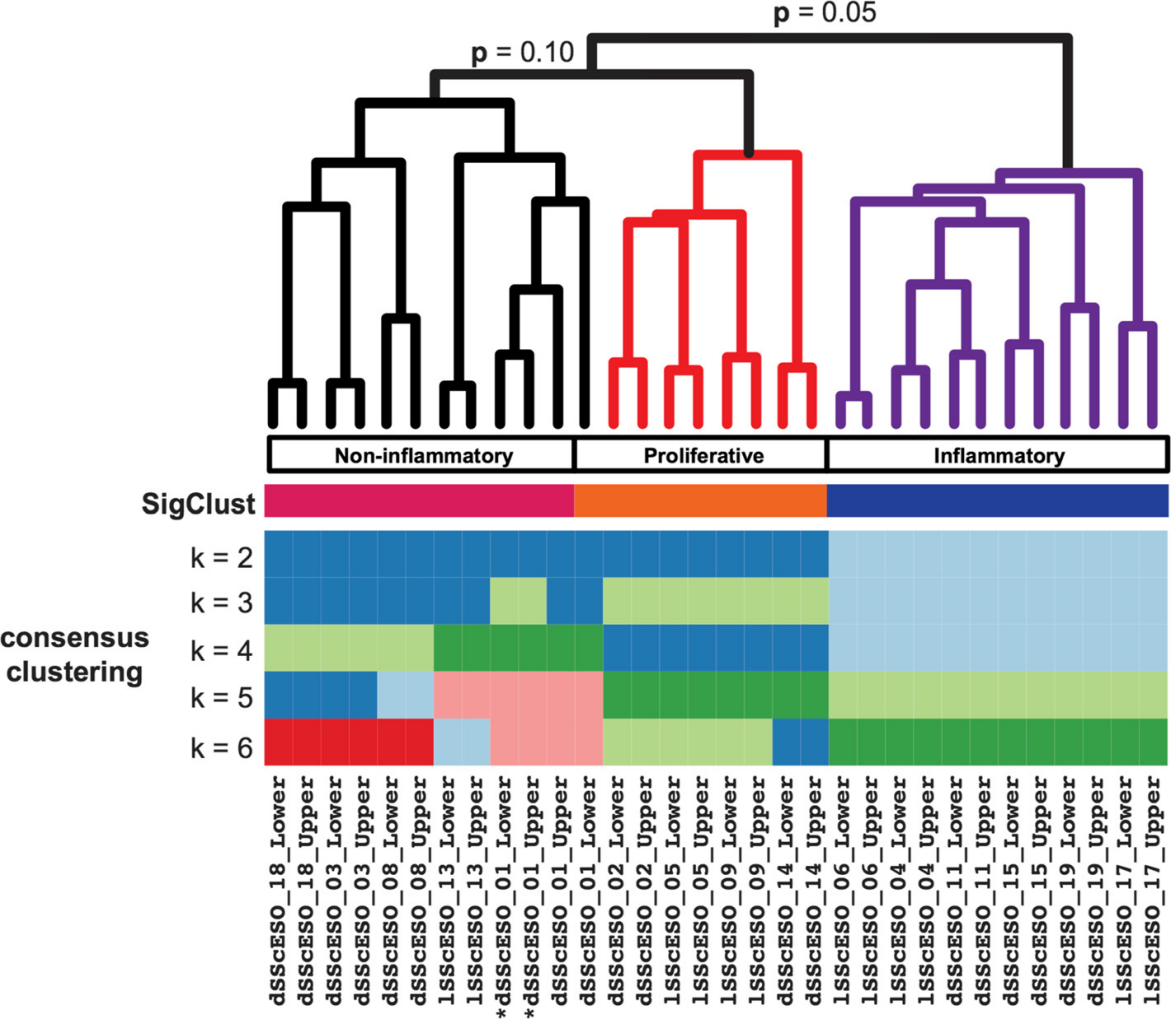
Esophageal biopsy cluster membership. Significance of clustering (SigClust) and consensus clustering assessed the robustness and significance of the sample clusters. **a** SigClust revealed two significant (*p* = 0.05) clusters of systemic sclerosis (SSc) patients. Biopsies from six patients demonstrated a stable inflammatory gene expression pattern. An *asterisk* indicates samples obtained at 6 months. The *colored bars* below the dendrogram indicate sample cluster membership from SigClust and consensus clustering. Additional file 10 contains the cumulative density function and delta area plots from the consensus clustering performed

To determine the statistical significance and stability of array clusters from intrinsic gene expression data, we employed consensus clustering and SigClust. SigClust analysis suggests that two to three distinct subsets exist in the patient cohort. The distinction between the inflammatory group and other biopsies was statistically significant (*p* = 0.05) (Fig. 2). SigClust analysis suggested two additional groups, termed proliferative and noninflammatory (*p* = 0.10). Analysis by consensus clustering, which performs multiple cluster analyses of different subsets of the data, demonstrates that the groups identified by SigClust largely cluster stably together with increasing *k* (Fig. 2 and Additional file 10). By focusing on the inflammatory, proliferative, and noninflammatory groups, the broad, generalizable biological differences based on the expression data are captured.

### Esophageal biopsies demonstrate inflammatory and proliferative molecular processes

**Fig. 3:**
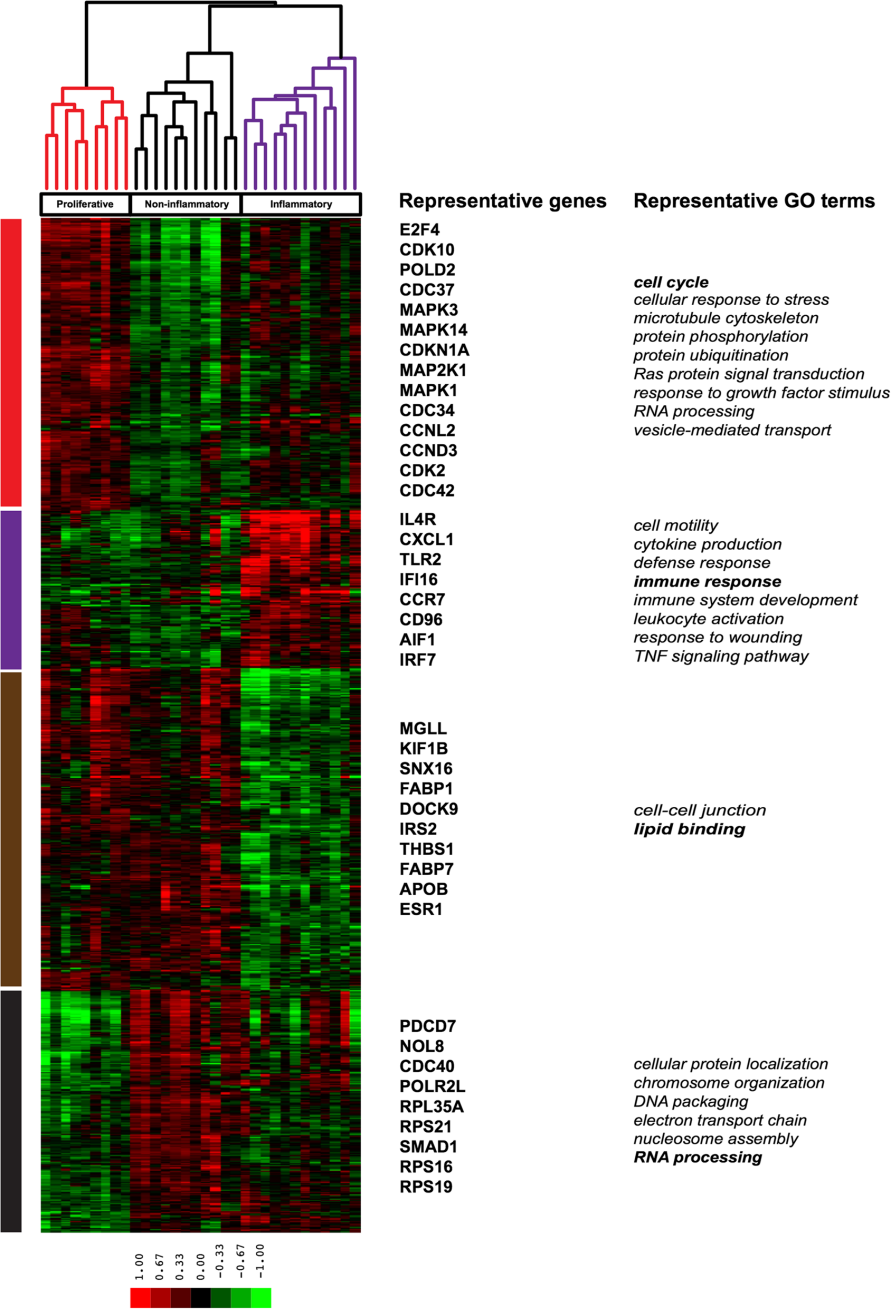
Functional enrichment analysis of genes differentially expressed between esophageal intrinsic subsets. Gene expression and functional enrichment in esophageal biopsies of systemic sclerosis (SSc) patients across three subsets as determined by significance of clustering (SigClust) (8490 probes, multiclass significance analysis of microarrays (SAM), false discovery rate (FDR) <1 %). Array tree legend: *red* arrays – samples from proliferative subset, *black* arrays – samples from noninflammatory subset, *purple* arrays – samples from inflammatory subset. Gene cluster legend: *red* cluster – genes and functional annotations upregulated in proliferative subset, *purple* cluster – genes and functional annotations upregulated in inflammatory subset, *brown* cluster – genes and functional annotations upregulated in proliferative and noninflammatory subsets, *black* cluster – genes and functional annotations upregulated in noninflammatory subset. Representative genes in bold are annotated to the GO term in bold. Additional file 12 contains a complete list of annotations from Additional file 8. Additional file 11 contains the full list of transcripts

To identify the molecular processes underlying the patient subsets, we analyzed the genes and pathways differentially expressed between patient groups. Significance Analysis of Microarrays (SAM) identified 8490 probes (5257 unique genes) differentially expressed between the three SSc groups (FDR <1 %) (Fig. 3 and Additional file 11). A total of 1317 probes (951 unique genes) showed increased expression in the inflammatory subset (Fig. 3, purple gene cluster) and enrichment in immune system activation (e.g. *immune response*, *p* = 2.40*10^−11^; *response to wounding*, *p* = 3.23*10^−12^; and *defense response*, *p* = 4.21*10^−07^). The proliferative subset showed 2448 probes (1748 unique genes) with increased expression (Fig. 3, red gene cluster). This group was enriched for cell cycle-related processes (e.g. *cell cycle*, *p* = 3.30*10^−10^; *cellular response to stress*, *p* = 2.75*10^−05^; *RNA processing*, *p* = 7.89*10^−05^). A total of 2023 probes (1166 unique genes) showed increased expression in the noninflammatory group (Fig. 3, black gene cluster). Functional enrichment analysis for this group of SSc patients identified chromosome organization and condensation (e.g. *chromosome organization*, *p* = 2.87*10^−05^; *nucleosome assembly*, *p* = 3.57*10^−05^; *DNA conformation change*, *p* = 2.49*10^−04^). A total of 2702 probes (1787 unique genes) showed decreased expression in the inflammatory SSc cluster and lack of coherent functional enrichment (Fig. 3, brown gene cluster). The complete functional enrichment results that accompany Fig. 3 are available in Additional file 12. Many of the genes and processes found here in esophagus strongly parallel those we find in SSc skin.

### qRT-PCR validation of microarray analysis

Selected genes with significant differences (*p* <0.001) in expression between patients from the inflammatory and proliferative intrinsic subsets were validated by qRT-PCR. Additional file 13 shows relative expression values normalized to the mean expression for upper and lower esophageal samples from a patient with normocytic anemia. Inflammatory patients demonstrated higher levels of *SOCS3* (*p* <0.01) and proliferative patients demonstrated higher levels of *CRISP2* (*p* <0.001) compared to the other group, respectively. Suppressor of cytokine signaling 3 (*SOCS3*) protein is a cytokine-inducible negative regulator of cytokine signaling, especially JAK2 kinase signaling [35]. The expression of *SOCS3* is induced by various cytokines, including interleukin (IL)-6 [36–38], IL-10 [39, 40], and interferon (IFN)-γ [41] that may be important in SSc. Cysteine-rich secretory protein 2 (*CRISP2*) is involved in cell-cell adhesion and is a member of the CAP superfamily of proteins that are thought to be important in immune function and cancer [42].

### Clinical and histopathological phenotypes associated with inflammatory esophageal biopsies

We compared the clinical, demographic and disease features as well as histopathological findings between the inflammatory group and the combined proliferative/noninflammatory group due to the significance and stability of the inflammatory subset (see Table 1 and Additional file 14). SSc patient biopsies clustered independently of SSc skin disease subtype (*p* = 0.62) (Fig. 1) and serum autoantibodies (*p* = 0.23) (see Table 1 and Additional file 14). Inflammatory patients were significantly older (*p* = 0.03) and had a positive smoking history although the difference was not statistically significant (*p* = 0.14). There was a trend toward more lung disease in the inflammatory group as evidenced by lower forced vital capacity (*p* = 0.13), total lung capacity (*p* = 0.13) and diffusion capacity for carbon monoxide percent predicted (*p* = 0.06) (Additional file 15). These findings demonstrate that patient subsets identified by gene expression are distinct from clinically defined subsets.

**Table 1:** Clinical variables

Mean (SD) or as indicated	Inflammatory group *N* = 6	Proliferative/noninflammatory group *N* = 9	*p* value^*^
Clinical variables			
Age	58.7 (8.3)	48.3 (7.7)	0.03
Sex, n (% women)	5 (83 %)	9 (100 %)	0.40
Ethnicity, n (% Caucasian)	5 (83 %)	5 (56 %)	0.58
BMI	22.4 (2.6)	24.5 (5.00)	0.30
Smoking, n (% past)	4 (67 %)	2 (22 %)	0.14
SSc subtype, n (% diffuse)	3 (50 %)	6 (67 %)	0.62
mRSS	12.7 (12.6)	16.6 (14.2)	0.59
GER symptoms, n (% present)	5 (83 %)	5 (56 %)	0.58
Dysphagia, n (% present)	3 (50 %)	3 (33 %)	0.62
Patulous esophagus HRCT, n (% present)	5 (83 %)	7 (78 %)	1.00
SSc disease duration (mo.)	153.7 (132.5)	83.7 (89.5)	0.29
GI symptom duration (mo.)	61.8 (62.2)	84.4 (87.0)	0.57
SSc autoantibodies, n (% positive)	N = 5		
• Scl-70	3 (50 %)	3 (33 %)	0.26
• ACA	1 (17 %)	0	
• RNA pol III	1 (17 %)	4 (44 %)	
• Negative	0	2 (22 %)	
• Missing	1 (17 %)	0	
Primary ANA pattern, n (% present)			
• Centromere	1 (17 %)	0	0.08
• Nucleolar	1 (17 %)	0	
• Speckled	1 (17 %)	7 (78 %)	
• Homogenous	3 (50 %)	2 (22 %)	
+ Mycophenolate, n (% current)	1 (17 %)	5 (56 %)	0.29
+Proton pump inhibition, N (% current)	6 (100 %)	9 (100 %)	N/A
FVC % predicted	70.2 (23.9)	88.9 (14.8)	0.13
TLC % predicted	81.8 (20.2)	100.6 (18.0)	0.13
DLCO % predicted	46.5 (17.5)	67.1 (20.3)	0.06
ILD present on HRCT, n (%)	5 (83 %)	7 (78 %)	1.00
Endoscopy, n (% present)			
Esophagitis	4 (67 %)	5 (56 %)	1.00
Hiatal hernia	5 (83 %)	7 (78 %)	1.00
Pathology			
Squamous epithelial lymphocytes	10.0 (8.7)	5.6 (5.9)	0.36
Basal cell hyperplasia, n (%)	N = 5	N = 7	
• 0	1 (17 %)	3 (33 %)	0.75
• 1	3 (50 %)	5 (56 %)	
• 2	1 (17 %)	0	
Degree of collagen deposition in lower esophageal biopsies, n (%)	N = 4	N = 4	
• 0	2 (33 %)	4 (44 %)	0.38
• 1	1 (17 %)	0	
• 2	1 (17 %)	0	
Candida esophagitis, n (%)	2 (33 %)	0	0.14

Only patients with SSc were included in these analyses. Subject SSc12 was excluded because the upper esophageal biopsy was influenced by technical artifact that precluded intrinsic subset assignment

*BMI* body mass index, *SSc* systemic sclerosis, *mRSS* modified Rodnan skin score, *GER* gastroesophageal reflux, *HRCT* high-resolution computed tomography, *GI* gastrointestinal, *Scl-70* anti-topoisomerase I, *ACA* anticentromere, *RNA pol III* anti-RNA polymerase III antibodies, *ANA* antinuclear antibodies, *N/A* not applicable, *FVC* forced vital capacity, *TLC* total lung capacity, *DLCO* diffusion capacity for carbon monoxide percent predicted, *ILD* interstitial lung disease

^*^ *t* test for continuous variables and Fisher’s exact test for categorical variables comparing inflammatory to proliferative/noninflammatory groups

### Inflammatory gene expression is independent of GER, collagen deposition and candida esophagitis

**Fig. 4:**
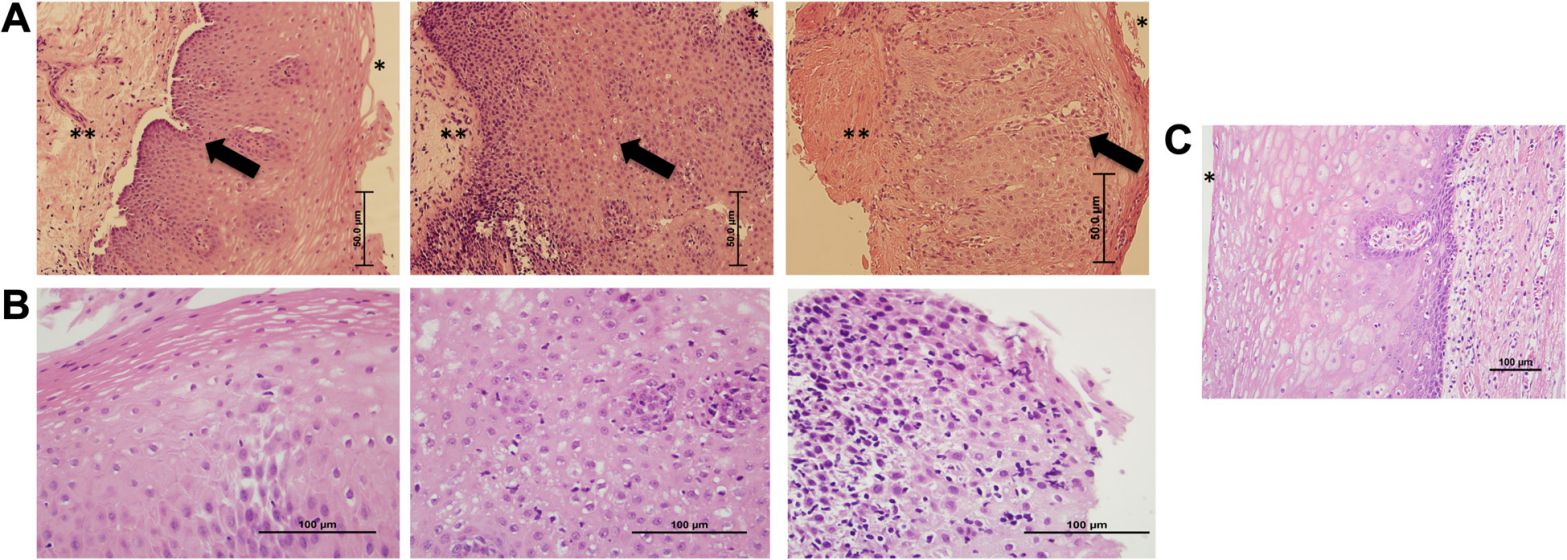
Systemic sclerosis (SSc) esophageal disease. **a**. Hematoxylin and eosin (H&E)-stained esophageal biopsies (20×) from patients with SSc representing stage 1, 2 and 3 fibrosis respectively (indicated as ^**^) and grade 0, 1, 2 basal cell hyperplasia in the lamina propria (*black arrow*). **b**. Representative photomicrographs (40×) of biopsy with <10 lymphocytes, 10–20 lymphocytes and >20 lymphocytes/high-power field (HPF) in the squamous epithelium. **c**. Esophageal biopsy (20×) from a healthy individual demonstrating no fibrosis, grade 0 basal cell hyperplasia and <10 lymphocytes/HPF. ^*^Indicates the esophageal lumen

Next, the association between histopathological phenotypes and esophageal gene expression signatures was evaluated for patients with SSc (Fig. 4). Importantly, all patients were receiving PPI (Additional file 1). Because GER, fungal infections and hiatal hernias can cause esophageal inflammation, we assessed whether inflammatory patients demonstrated more GER- or candida-associated histopathological changes and/or hiatal hernias. On EGD, gross evidence for esophagitis and/or hiatal hernias was present in five out of six (83 %) inflammatory and seven out of nine (78 %) patients classified in the heterogeneous group (*p* = 1.00) (Table 1 and Additional file 14). Evidence for candida infection was present upon H&E-stained biopsies for two SSc patients both of whom were classified in the inflammatory subset (Additional file 14). Next, a GI pathologist scored esophageal biopsies for degree of basal cell hyperplasia and the number of intraepithelial lymphocytes, both GER markers (Fig. 4). In biopsies from the lower esophagus, the degree of basal cell hyperplasia was 11 % grade 0, 33 % grade 1, and 56 % grade 2 for inflammatory patients, and 33 % grade 0, 56 % grade 1, and 0 grade 2 for heterogeneous subjects (*p* = 0.75) (Fig. 4a). The mean ± SD number of squamous epithelial lymphocytes in inflammatory patient biopsies was 10.0 ± 8.7 compared to 5.6 ± 5.9 in biopsies from patients classified in the heterogeneous group (*p* = 0.36) (Fig. 4b). These data suggest that the presence of the inflammatory gene expression signature in lower esophageal biopsies was unrelated to reflux or candida esophagitis either by endoscopic or histological criteria.

Next, we examined the concordance between upper and lower esophageal biopsies for GER evidence. Based upon basal cell hyperplasia, no control subjects and two SSc patients had upper GERD: two control subjects and one SSc patient had lower GERD. Based upon lymphocyte counts, no control or SSc patient had upper GERD: one control subject and one SSc patient had lower GERD. Importantly, there were SSc patients that had evidence for GERD in upper biopsies with normal lower biopsies. Based upon these data, histological evidence for GERD is definition-dependent; histological findings of upper and lower GERD may lack concordance in individuals; and upper GERD does not appear to be more prevalent in SSc patients compared to non-SSc patients, but the numbers of subjects was small.

Lastly, we scored lower esophageal biopsies for degree of collagen deposition in the lamina propria (Fig. 4a). There was no difference in collagen deposition between inflammatory (33 % grade 0, 17 % grade 1, 17 % grade 2) and proliferative/noninflammatory patients (44 % grade 0, 0 % grade 1, 0 % grade 2) (*p* = 0.38) (Table 1 and Additional file 14).

## Discussion

Esophageal dysmotility and dysphagia cause considerable morbidity in patients with SSc. Despite its prevalence, the pathogenesis is poorly understood in large part because smooth muscle cells dedifferentiate in culture. Moreover, no animal models of scleroderma esophageal disease, no biomarkers for disease progression, and no disease-modifying treatments have been identified. Patients with SSc routinely undergo EGD with esophageal biopsies for clinical indications. We performed a molecular characterization of gene expression combined with detailed histological analyses of esophageal biopsies to identify biomarkers of esophageal dysfunction and increase our understanding of SSc esophageal disease pathogenesis.

We identified robust molecular subsets of SSc esophageal disease that are distinct from clinically determined subtypes. A subset of patients with SSc esophageal disease had an inflammatory gene expression signature while another group had a proliferative/noninflammatory signature. We showed that these signatures appear to be independent of traditional clinical markers of SSc including disease subtype and duration, serum autoantibodies and skin score, but the sample size is small. A patulous esophagus on HRCT, esophagitis and/or hiatal hernia on EGD, PFT reductions and immune modulating medication use were not different between groups. With the exception of older age in the patients expressing esophageal inflammation, there were no significant clinical differences between patients in the inflammatory and the proliferative/noninflammatory groups. Importantly, all patients in both groups were taking stable doses of PPIs.

Our results demonstrate that SSc intrinsic gene expression subsets are present in esophagus as well as in skin. Three subsets (inflammatory, proliferative and noninflammatory) were identified in esophageal biopsies compared to four intrinsic subsets identified in skin biopsies. This observation suggests that we are witnessing inherent heterogeneity in the SSc patient population. The addition of more SSc esophageal samples into this dataset may reveal additional important subsets. For example, the noninflammatory group may be subdivided into two groups as indicated by consensus clustering.

Our findings are significant because they demonstrate that although end-target tissues in SSc display molecular heterogeneity, there are robust gene expression patterns and molecular pathways that are conserved across tissues. It has been observed that molecular signatures from the same disease in different tissues are more similar than gene expression from different diseases in the same tissue and may allow for the construction of multi-tissue models of pathogenesis [43]. Our results suggest that the strong inflammatory signal, and possibly the proliferative signature, seen in skin and now in the esophagus reflect common pathogenic processes in SSc.

Importantly, there was no evidence that inflammation resulting from underlying hiatal hernia or GER and candida esophagitis drove the esophageal inflammatory gene expression signature. The prevalence of GER and candida esophagitis and hiatal hernia on EGD, increased basal cell hyperplasia and intraepithelial lymphocytes, and proton pump inhibitor use were not statistically different between SSc patients who did or did not express the inflammatory gene expression signature though the numbers are small. If the inflammatory signature was GER-dependent, we would expect the lower esophageal biopsies from patients in the inflammatory subset to cluster together and separately from the upper biopsies. However, this was not observed (Additional file 7): upper and lower biopsies from the same patient clustered side by side. Further, the nonsignificant difference in lymphocytes present between the two groups suggests that part of the observed inflammatory gene expression signature is due to lymphocyte activation and may also be driven by the infiltration of other cell types such as macrophages or eosinophils.

On a practical level, the feasibility of conducting molecular studies on esophageal tissue in patients with SSc was established. No subjects experienced any EGD complications, and patients were willing to undergo multiple esophageal biopsies for clinical and research purposes. Esophageal biopsies from healthy control subjects were not obtained, which is a limitation of our current study. Because collagen is a normal esophageal component and not necessarily indicative of fibrosis, future studies should include deeper biopsies to ensure lamina propria sampling. Lamina propria was sampled in 30 % of esophageal biopsies included in the present study, limiting the conclusions that can be drawn. Esophageal functional studies were also not performed in the majority of patients. Prospective studies are underway to identify associations between gene expression, esophageal histology and changes and esophageal function in SSc patients and healthy control subjects.

## Conclusions

Biomarker identification and targeted treatment development for esophageal disease in SSc represent a large unmet clinical need. We are the first to employ whole-genome gene expression analyses of esophageal biopsies from patients with SSc to gain insights into their esophageal disease pathogenesis. We identified inflammatory and proliferative/noninflammatory gene expression signatures in SSc esophageal biopsies that appear to be independent of clinical markers of SSc disease as well as medication use though the sample size is small. Importantly, inflammatory and proliferative/noninflammatory gene expression signatures that were previously identified in SSc skin were recapitulated in SSc esophageal biopsies. This finding suggests that the overarching deregulated molecular programs responsible for SSc are similar in different end organs. Studies are underway to determine the concordance between skin and esophageal gene expression signatures for individual patients. Lastly, we demonstrate the utility and feasibility of genome-wide analyses of gene expression in esophageal biopsies from SSc patients. Efforts are underway to further analyze these data to identify new treatment targets as well as currently available medications that can be repurposed to treat SSc esophageal dysmotility. Results of gene expression analysis of tissues from patients with SSc hold promise for individualized patient care that permits treatment selection based upon knowledge of deregulated molecular pathways.

## Additional files

Additional file 1:
**Subjects and biopsy time points.**
Additional file 2:
**gPCA analysis of SSc samples.** gPCA (R package v1.0) provides a statistical test for identifying batch bias in high-throughput genomic data (18). (*A*) Scatterplots and density plots of the first, second, and third principal components from guided PCA before batch correction with ComBat demonstrates batch bias, *p* value <0.001 and (*B*) after batch correction. ComBat removes batch bias, *p* value = 0.997. Additional file 3:
**Human qRT-PCR primers (5′-3′).**
Additional file 4:
**Differential gene expression analysis between controls and SSc patients.** A total of 1063 probes were found to be differentially expressed between control and SSc samples (*p* <0.05). (*A*) Array tree structure. *Green labels and edges* indicate controls. *Black edges* indicate SSc patients. *Black labels* indicate patients with lSSc and *red labels* indicate patients with dSSc. An *asterisk* indicates samples obtained at 6 months. (*B*) Overview of gene expression patterns. Additional file 5.
**Expression data matrices. All matrices are supplied as tab-delimited text files.** (*1*) Expression data for 32 arrays (all probes). Input to intrinsic gene analysis and SAM (Fig. 3). (*2*) Expression data for 32 arrays (intrinsic genes, FDR <1.1 %). Used for Figs. 1 and 2. (*3*) Thirty-three arrays, SSc only (all probes). Used for Figure S4 in Additional file 7. (*4*) Expression data for 43 arrays, no controls with autoimmune disorders (all probes). Used for Figure S2 in Additional file 6. Following normalization and log-transformation, each version of the dataset was processed in the following way: arrays not being considered were removed. Missing values were imputed using a *k*-nearest neighbor algorithm. Nonparametric ComBat was used to adjust batch effects. Genes were median-centered. Additional file 6:
**Differential gene expression analysis between controls and SSc patients.** A total of 1903 probes (1350 unique genes) were found to be differentially expressed between control and SSc samples (*p* <0.05). (*A*) Array tree structure. *Green labels and edges* indicate controls. *Black edges* indicate SSc patients. *Black labels* indicate patients with lSSc and *red labels* indicate patients with dSSc. An *asterisk* indicates samples obtained at 6 months. Brackets indicate biopsies from the upper and lower esophagus for an individual that clustered together. (*B*) Overview of gene expression patterns. *Top green bar* indicates a group of genes with expression patterns similar between controls and a subset of SSc patients including patients 2, 5, 9 and 14. *Bottom red bar* indicates a group of genes with expression patterns similar between controls and a subset of SSc patients including patients 4, 11, 15, 17 and 19. Additional file 7:
**Gene expression in esophageal biopsies from patients with SSc.** (*A*) Dendrogram of hierarchical clustering of samples based on 3507 probes identified as present in ≥2 arrays with values ≥2-fold change over median. *Brackets* indicate biopsies from the upper and lower esophagus for an individual that clustered together. SSc patient biopsies clustered independently of lcSSc and dcSSc designation shown in *black* and *red*, respectively. An *asterisk* indicates samples obtained at 6 months. (B) Overview of hierarchically clustered probes. (*C*) A subset of SSc patients shows overexpression of an inflammatory gene signature (*blue and purple clusters*). The leaves of the dendrogram indicating the inflammatory subset of arrays are shown in *purple*. Additional file 8:
**Genes differentially expressed between paired upper and lower SSc esophageal biopsies.** Comparative Marker Selection was used to perform a paired *t* test comparing patients’ upper and lower biopsies. Differentially expressed transcripts (1479 probes; FDR <5 %) were selected and arrays were hierarchically clustered. Upper and lower biopsies cluster side by side in 14 out of 16 patients. An *asterisk* indicates samples obtained at 6 months. *Brackets* indicate biopsies from the upper and lower esophagus for an individual that clustered together. Additional file 9:
**Intrinsic gene analysis, transcripts with FDR <1.1 %.** This figure is intended to be viewed digitally and includes probe IDs and annotations for all transcripts included in Fig. 1. Additional file 10:
**Consensus clustering of intrinsic gene data.** The consensus cumulative density function (CDF) and delta area plots of the different numbers of clusters tested in consensus clustering. The number of clusters present in the data is identified when the area under the CDF curve does not increase greatly between *k*, or there is no proportional increase in area between increasing *k* as visualized in the delta plot. Additional file 11:
**Functional analysis of intrinsic subsets.** This figure is intended to be viewed digitally and includes probe IDs and annotations for all transcripts included in Fig. 3. Additional file 12:
**Complete g:Profiler results from genes differentially expressed between SSc groups (Fig.** 4 **).**
Additional file 13:
**Quantitative polymerase chain reaction validation of microarray results.** Values are normalized to ESO3. Additional file 14:
**Esophageal histological scoring.**
Additional file 15:
**Clinical covariates, dichotomous cluster stripcharts.** Bars represent mean with 95 % CI.

## Abbreviations

ANA: antinuclear antibodies
dcSSc: diffuse cutaneous SSc
EGD: esophagogastroduodenoscopy
FDR: false discovery rate
GER: gastroesophageal reflux
GI: gastrointestinal
H&E: hematoxylin and eosin
HPF: high-power field
HRCT: high-resolution computed tomography
IFN: interferon
IL: interleukin
ILD: interstitial lung disease
lcSSc: limited cutaneous SSc
mRSS: modified Rodnan skin score
PFT: pulmonary function tests
PPI: proton pump inhibition
qRT-PCR: quantitative reverse transcriptase-polymerase chain reaction
SAM: significance analysis of microarrays
SigClust: significance of clustering
SSc: systemic sclerosis (scleroderma)
