# Supplementary material for: Molecular characterization of systemic sclerosis esophageal pathology identifies inflammatory and proliferative signatures
Source: Arthritis Res Ther. 2015 Jul 29;17:194. doi: 10.1186/s13075-015-0695-1 (PMC4518531; doi:10.1186/s13075-015-0695-1)
Supplement: Additional file 1: — Subjects and biopsy time points. [file 13075_2015_695_MOESM1_ESM.pdf]

Table S1: Subjects and biopsy time points

Study Code	EGD Indication	Age/Sex	Ethnicity	Diagnosis	PPI duration (months)	Current PPI	SSc therapy	Scl-70	RNA Pol III	ACA
Eso1	dysphagia	50/F	White	Schatzki's ring	0	None	N/A	N/A	N/A	N/A
Eso2	heartburn, upper abdominal pain	78/F	White	Lupus with GER	95	dexlansoprazole	N/A	N/A	N/A	N/A
Eso3	iron deficiency anemia	45/F	Black	Anemia	0	None	N/A	N/A	N/A	N/A
Eso4	nausea with vomiting	24/M	Hispanic	GER	0	None	N/A	N/A	N/A	N/A
Eso5	epigastric abdominal pain, weight loss	31/F	White	SA with GER	66	esomeprazole	N/A	N/A	N/A	N/A
Eso6	GER follow-up, intestinal metaplasia of gastroesophageal junction	34/M	White	GER	156	omeprazole	N/A	N/A	N/A	N/A
Eso7	heartburn, dysphagia	24/F	White	GER	84	omeprazole	N/A	N/A	N/A	N/A
SScEso1*	refractory GER abdominal pain	52/F	White	dSSc	40	dexlansoprazole	Stem Cell	-	+	-
SScEso2	abdominal pain, dysphagia	30/F	Hispanic	dSSc	22	omeprazole	None	+	N/A	-
SScEso3	dysphagia	49/F	White	dSSc	32	esomeprazole	MMF	-	+	-
SScEso4	dysphagia	53/F	White	lSSc	60	esomeprazole	None	-	-	+
SScEso5	GER	58/F	White	lSSc	16	lansoprazole	None	-	-	-
SScEso6	heartburn, dysphagia, regurgitation	63/M	White	lSSc	57	dexlansoprazole lansoprazole	MMF	-	N/A	-
SScEso8	heartburn, dysphagia	50/F	Asian	dSSc	25	lansoprazole	MMF	-	-	-
SScEso9	dysphagia, GER	52/F	White	lSSc	97	dexlansoprazole	None	+	-	-
SScEso11	heartburn, dysphagia, anemia, weight loss	68/F	White	dSSc	13	pantoprazole	None	+	-	-
SScEso12	gastric arteriovenous malformation	49/F	White	dSSc	4	pantoprazole	MMF	-	+	-
SScEso13	heartburn, dysphagia	47/F	White	lSSc	289	omeprazole	MMF	+	-	-
SScEso14	iron deficiency anemia, GAVE	46/F	Mixed	dSSc	23	omeprazole	MMF	-	+	-

SScEso15	left upper quadrant abdominal pain established GER	50/F	Hispanic	dSSc	87	dexlansoprazole	None	-	+	-
SScEso17	heartburn, GER follow-up	67/F	White	lSSc	118	esomeprazole	None	+	-	-
SScEso18	heartburn, dysphagia, iron deficiency anemia	51/F	Black	dSSc	48	esomeprazole	MMF	-	+	-
SScEso19	dysphagia	51/F	White	dSSc	96	esomeprazole	None	+	-	-

EGD=esophagogastroduodenoscopy, PPI=proton pump inhibition, SSc=systemic sclerosis, Scl-70=anti-topoisomerase I, RNA Pol III=RNA polymerase III, ACA=anticentromere serum autoantibodies, GER=gastroesophageal reflux disease, SA=spondyloarthropathy, dSSc=diffuse cutaneous, lSSc=limited cutaneous SSc, N/A=not available, MMF=mycophenolate mofetil. *indicates baseline and 6mo sample obtained.
