# Supplementary figures and images for: Molecular characterization of systemic sclerosis esophageal pathology identifies inflammatory and proliferative signatures
Source: Arthritis Res Ther. 2015 Jul 29;17:194. doi: 10.1186/s13075-015-0695-1 (PMC4518531; doi:10.1186/s13075-015-0695-1)

A

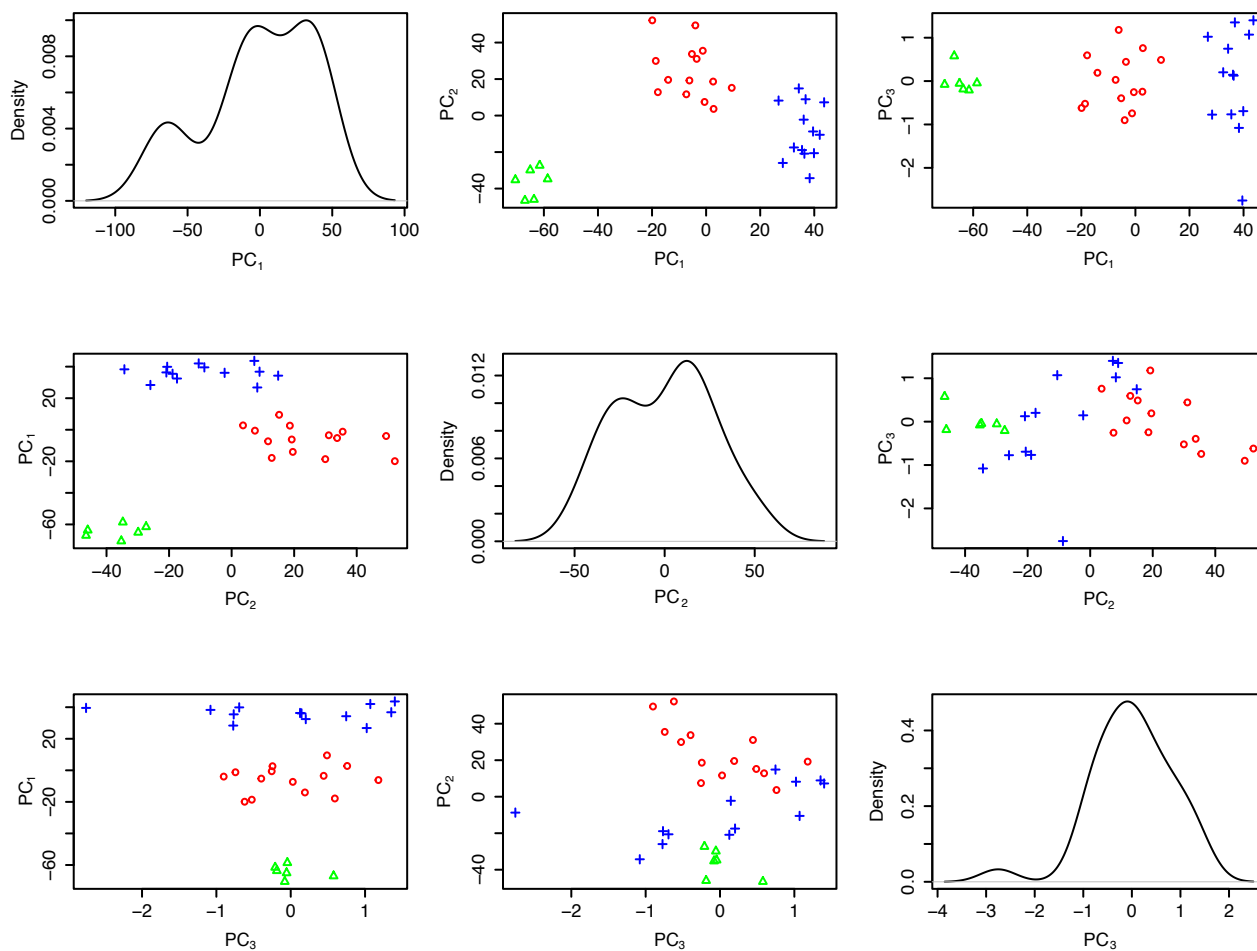

B

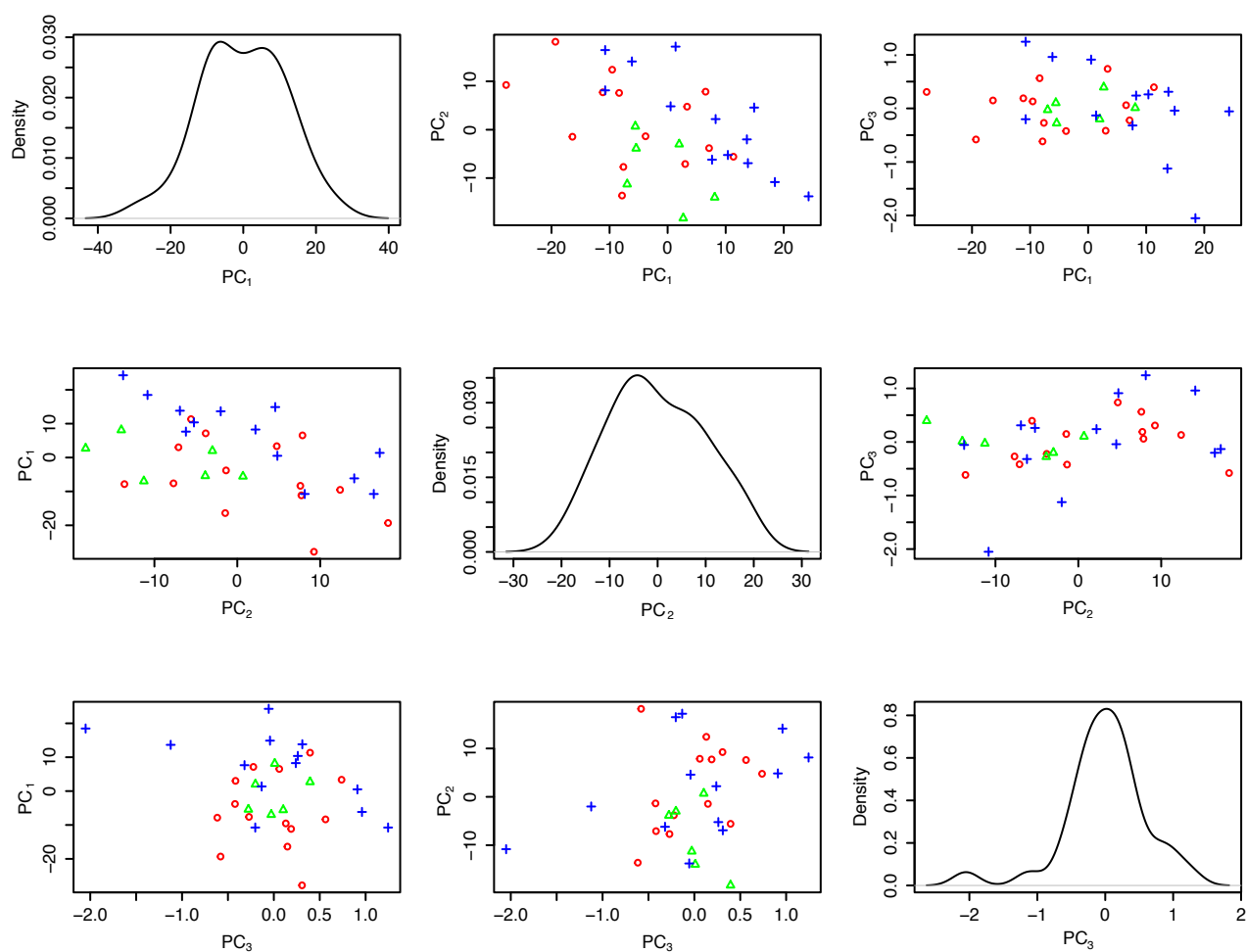

Supplement: Additional file 2: — gPCA analysis of SSc samples. gPCA (R package v1.0) provides a statistical test for identifying batch bias in high-throughput genomic data (18). (A) Scatterplots and density plots of the first, second, and third principal components from guided PCA before batch correction with ComBat demonstrates batch bias, p value <0.001 and (B) after batch correction. ComBat removes batch bias, p value = 0.997. [file 13075_2015_695_MOESM2_ESM.pdf]

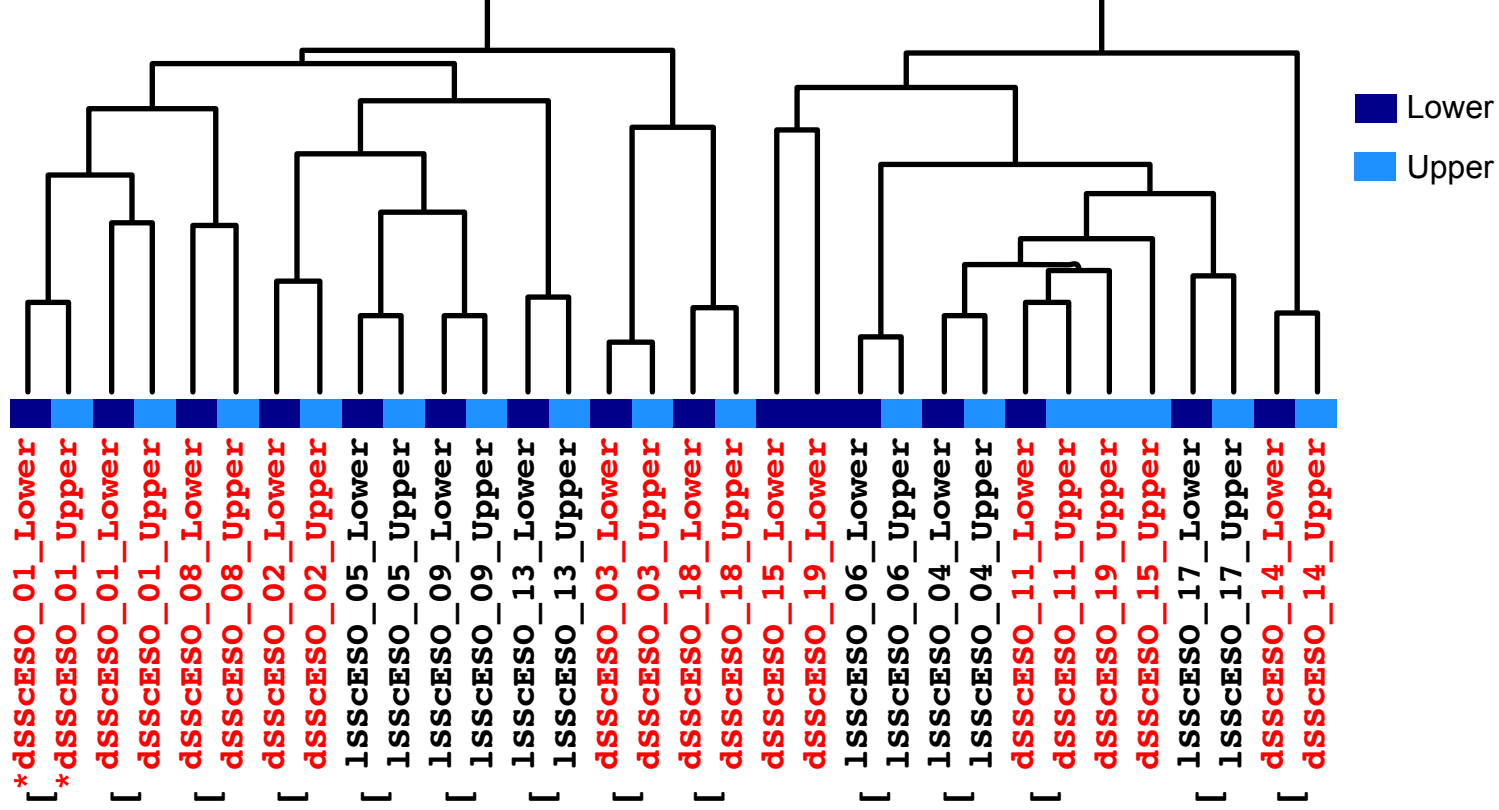

Supplement: Additional file 8: — Genes differentially expressed between paired upper and lower SSc esophageal biopsies. Comparative Marker Selection was used to perform a paired t test comparing patients’ upper and lower biopsies. Differentially expressed transcripts (1479 probes; FDR <5 %) were selected and arrays were hierarchically clustered. Upper and lower biopsies cluster side by side in 14 out of 16 patients. An asterisk indicates samples obtained at 6 months. Brackets indicate biopsies from the upper and lower esophagus for an individual that clustered together. [file 13075_2015_695_MOESM8_ESM.pdf]

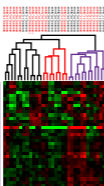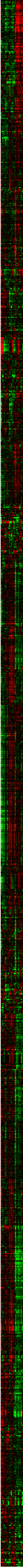

Supplement: Additional file 9: — Intrinsic gene analysis, transcripts with FDR <1.1 %. This figure is intended to be viewed digitally and includes probe IDs and annotations for all transcripts included in Fig. 1. [file 13075_2015_695_MOESM9_ESM.pdf]

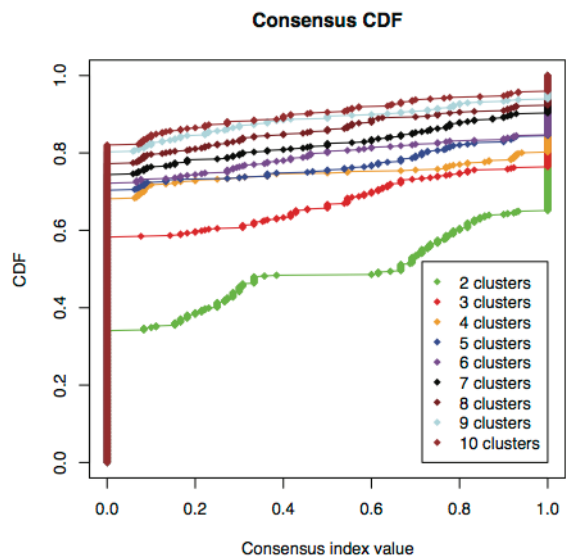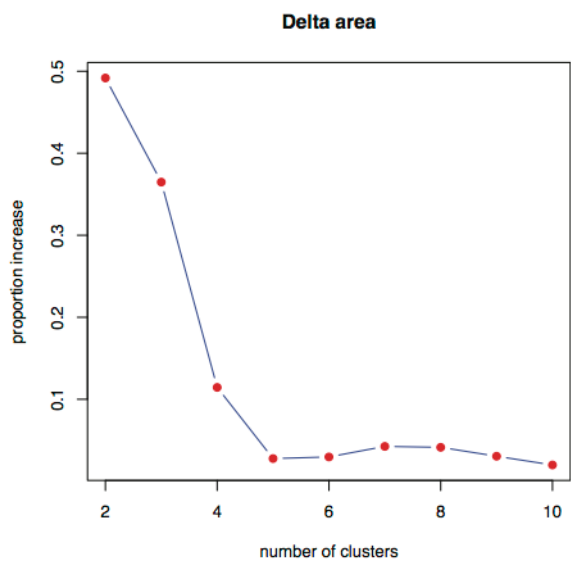

Supplement: Additional file 10: — Consensus clustering of intrinsic gene data. The consensus cumulative density function (CDF) and delta area plots of the different numbers of clusters tested in consensus clustering. The number of clusters present in the data is identified when the area under the CDF curve does not increase greatly between k, or there is no proportional increase in area between increasing k as visualized in the delta plot. [file 13075_2015_695_MOESM10_ESM.pdf]

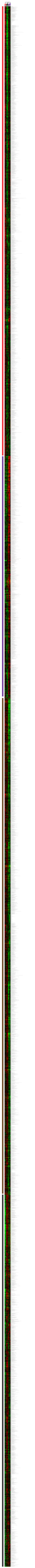

Supplement: Additional file 11: — Functional analysis of intrinsic subsets. This figure is intended to be viewed digitally and includes probe IDs and annotations for all transcripts included in Fig. 3. [file 13075_2015_695_MOESM11_ESM.pdf]

### CRISP2

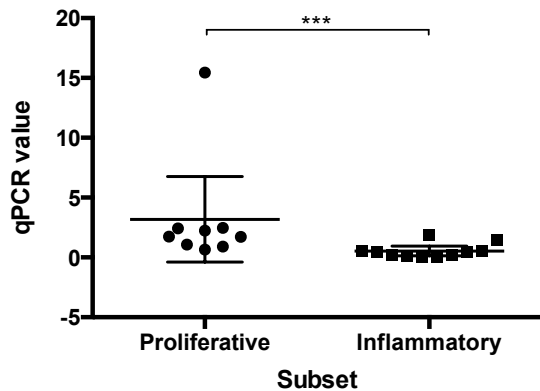

### CRISP2

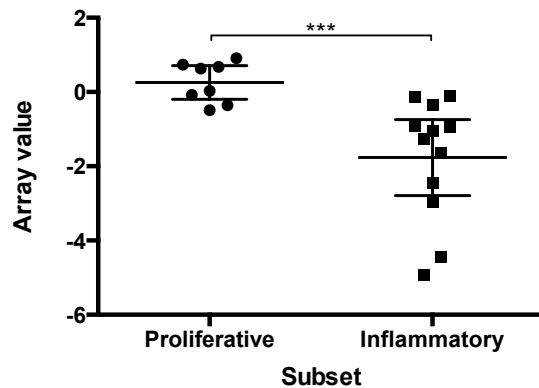

### SOCS3

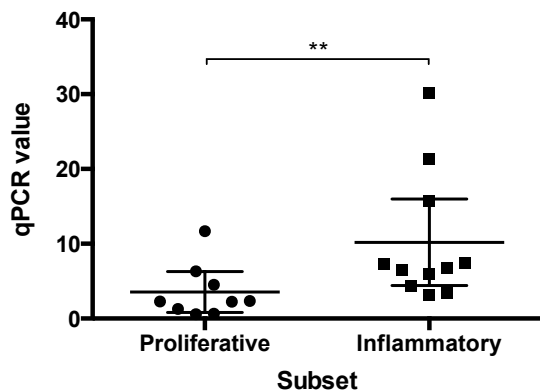

### SOCS3

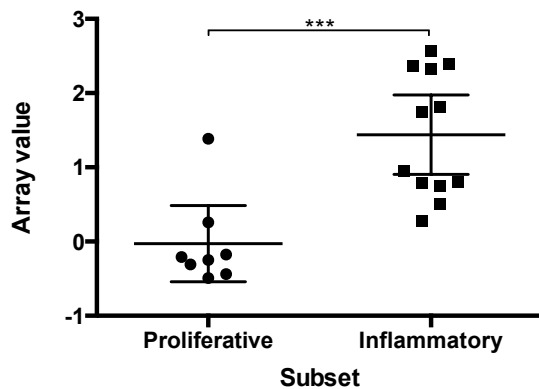

Supplement: Additional file 13: — Quantitative polymerase chain reaction validation of microarray results. Values are normalized to ESO3. [file 13075_2015_695_MOESM13_ESM.pdf]

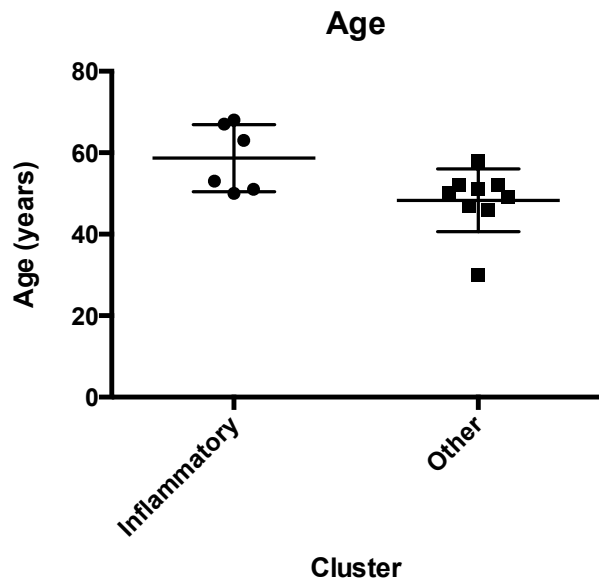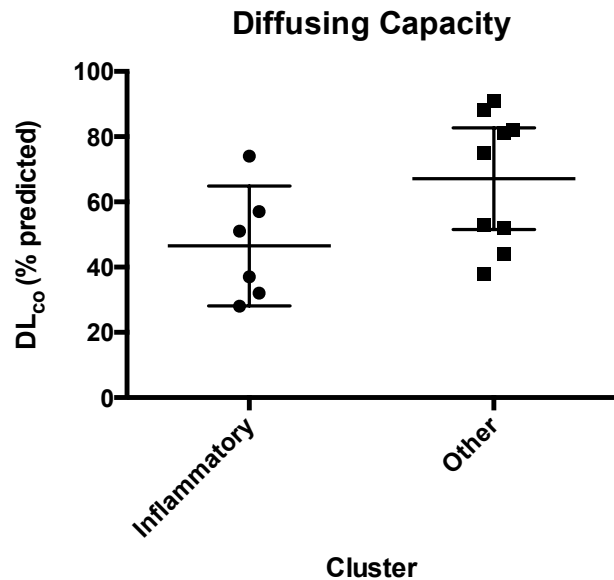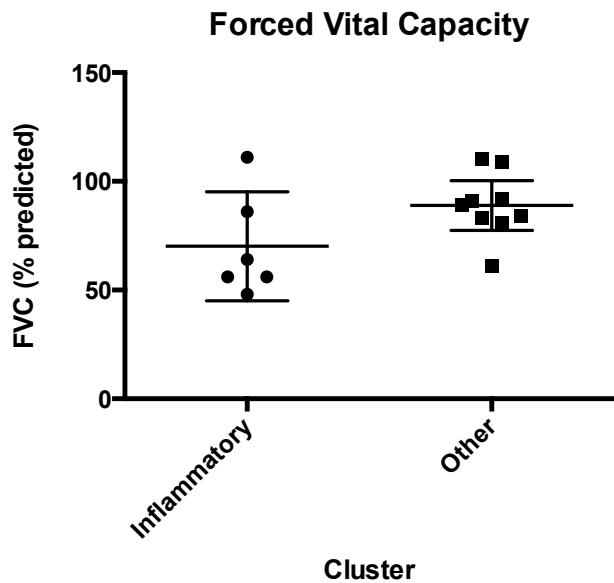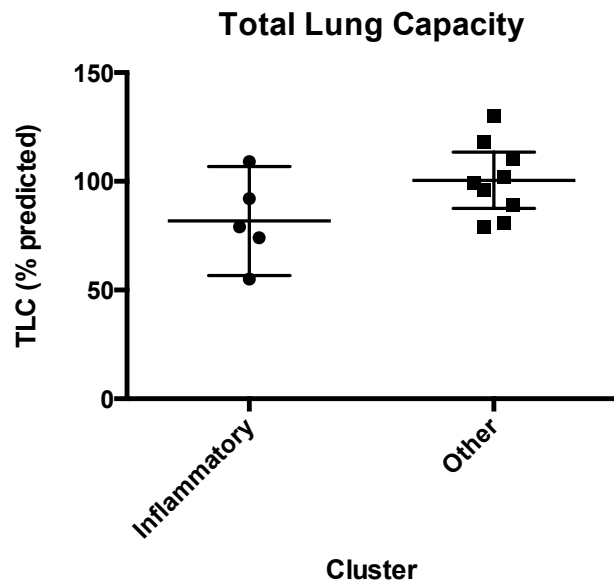

Supplement: Additional file 15: — Clinical covariates, dichotomous cluster stripcharts. Bars represent mean with 95 % CI. [file 13075_2015_695_MOESM15_ESM.pdf]
