# Supplementary material for: Molecular characterization of systemic sclerosis esophageal pathology identifies inflammatory and proliferative signatures
Source: Arthritis Res Ther. 2015 Jul 29;17:194. doi: 10.1186/s13075-015-0695-1 (PMC4518531; doi:10.1186/s13075-015-0695-1)
Supplement: Additional file 3: — Human qRT-PCR primers (5′-3′). [file 13075_2015_695_MOESM3_ESM.pdf]

Table S2: Human qRT-PCR primers (5'-3')

SOCS3	Forward	CCTGCGCCTCAAGACCTTC
	Reverse	GTCACTGCGCTCCAGTAGAA
CRISP2	Forward	GGAGCAGAGAGGTAACAACGA
	Reverse	TTGTACTGGTTTTGCGGTCCT
