# Supplementary material for: Molecular characterization of systemic sclerosis esophageal pathology identifies inflammatory and proliferative signatures
Source: Arthritis Res Ther. 2015 Jul 29;17:194. doi: 10.1186/s13075-015-0695-1 (PMC4518531; doi:10.1186/s13075-015-0695-1)
Supplement: Additional file 4: — Differential gene expression analysis between controls and SSc patients. A total of 1063 probes were found to be differentially expressed between control and SSc samples (p <0.05). (A) Array tree structure. Green labels and edges indicate controls. Black edges indicate SSc patients. Black labels indicate patients with lSSc and red labels indicate patients with dSSc. An asterisk indicates samples obtained at 6 months. (B) Overview of gene expression patterns. [file 13075_2015_695_MOESM4_ESM.pdf]

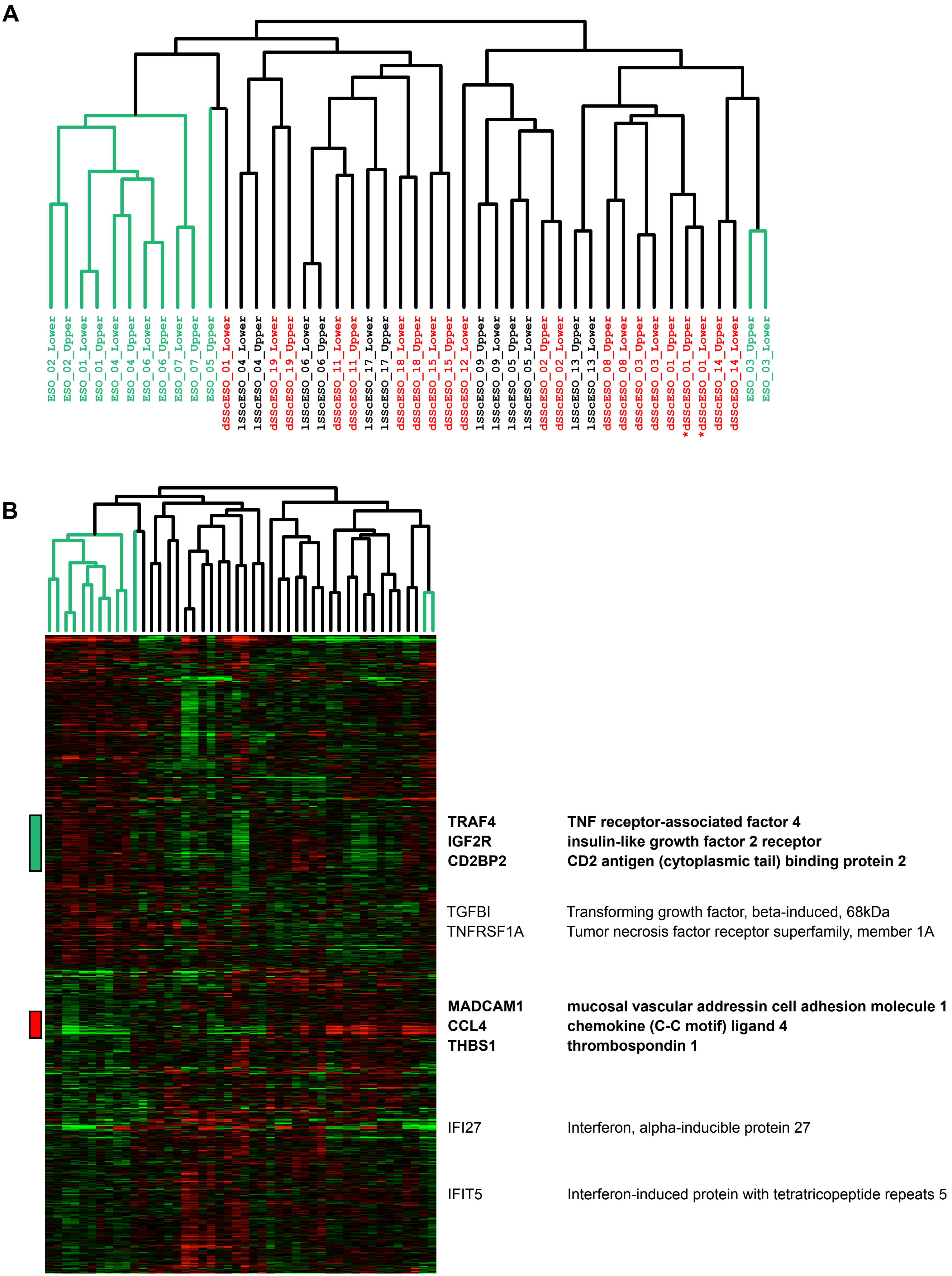
A
ESO_02_Lower
ESO_02_Upper
ESO_01_Lower
ESO_01_Upper
ESO_04_Lower
ESO_04_Upper
ESO_06_Lower
ESO_06_Upper
ESO_07_Lower
ESO_07_Upper
ESO_05_Upper
dSScESO_01_Lower
lSScESO_04_Lower
lSScESO_04_Upper
dSScESO_19_Lower
dSScESO_19_Upper
lSScESO_06_Lower
lSScESO_06_Upper
dSScESO_11_Lower
dSScESO_11_Upper
lSScESO_17_Lower
lSScESO_17_Upper
dSScESO_18_Lower
dSScESO_18_Upper
dSScESO_15_Lower
dSScESO_15_Upper
dSScESO_12_Lower
lSScESO_09_Upper
lSScESO_09_Lower
lSScESO_05_Upper
lSScESO_05_Lower
dSScESO_02_Upper
dSScESO_02_Lower
lSScESO_13_Upper
lSScESO_13_Lower
dSScESO_08_Upper
dSScESO_08_Lower
dSScESO_03_Upper
dSScESO_03_Lower
dSScESO_01_Upper
*dSScESO_01_Upper
*dSScESO_01_Lower
dSScESO_14_Upper
dSScESO_14_Lower
ESO_03_Upper
ESO_03_Lower
B
TRAF4 TNF receptor-associated factor 4
IGF2R insulin-like growth factor 2 receptor
CD2BP2 CD2 antigen (cytoplasmic tail) binding protein 2
TGFBI Transforming growth factor, beta-induced, 68kDa
TNFRSF1A Tumor necrosis factor receptor superfamily, member 1A
MADCAM1 mucosal vascular addressin cell adhesion molecule 1
CCL4 chemokine (C-C motif) ligand 4
THBS1 thrombospondin 1
IFI27 Interferon, alpha-inducible protein 27
IFIT5 Interferon-induced protein with tetratricopeptide repeats 5
