# Supplementary material for: Molecular characterization of systemic sclerosis esophageal pathology identifies inflammatory and proliferative signatures
Source: Arthritis Res Ther. 2015 Jul 29;17:194. doi: 10.1186/s13075-015-0695-1 (PMC4518531; doi:10.1186/s13075-015-0695-1)
Supplement: Additional file 6: — Differential gene expression analysis between controls and SSc patients. A total of 1903 probes (1350 unique genes) were found to be differentially expressed between control and SSc samples (p <0.05). (A) Array tree structure. Green labels and edges indicate controls. Black edges indicate SSc patients. Black labels indicate patients with lSSc and red labels indicate patients with dSSc. An asterisk indicates samples obtained at 6 months. Brackets indicate biopsies from the upper and lower esophagus for an individual that clustered together. (B) Overview of gene expression patterns. Top green bar indicates a group of genes with expression patterns similar between controls and a subset of SSc patients including patients 2, 5, 9 and 14. Bottom red bar indicates a group of genes with expression patterns similar between controls and a subset of SSc patients including patients 4, 11, 15, 17 and 19. [file 13075_2015_695_MOESM6_ESM.pdf]

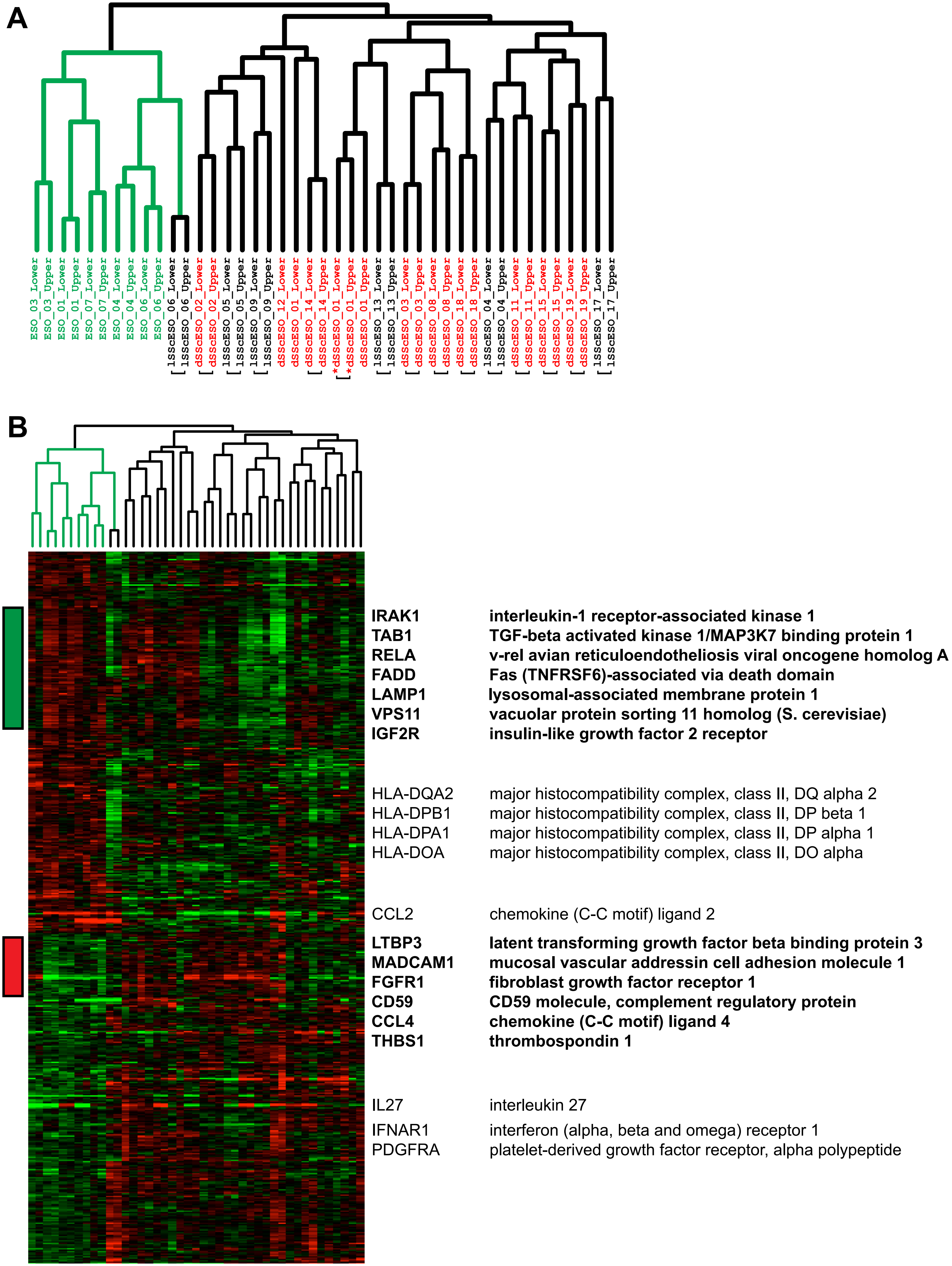
A
ESO_03_Lower
ESO_03_Upper
ESO_01_Lower
ESO_01_Upper
ESO_07_Lower
ESO_07_Upper
ESO_04_Lower
ESO_04_Upper
ESO_06_Lower
ESO_06_Upper
lSScESO_06_Lower
lSScESO_06_Upper
dSScESO_02_Lower
dSScESO_02_Upper
lSScESO_05_Lower
lSScESO_05_Upper
lSScESO_09_Lower
lSScESO_09_Upper
dSScESO_12_Lower
dSScESO_01_Lower
dSScESO_14_Lower
dSScESO_14_Upper
*dSScESO_01_Lower
*dSScESO_01_Upper
dSScESO_01_Upper
lSScESO_13_Lower
lSScESO_13_Upper
dSScESO_03_Lower
dSScESO_03_Upper
dSScESO_08_Lower
dSScESO_08_Upper
dSScESO_18_Lower
dSScESO_18_Upper
lSScESO_04_Lower
lSScESO_04_Upper
dSScESO_11_Lower
dSScESO_11_Upper
dSScESO_15_Lower
dSScESO_15_Upper
dSScESO_19_Lower
dSScESO_19_Upper
lSScESO_17_Lower
lSScESO_17_Upper
B
IRAK1 interleukin-1 receptor-associated kinase 1
TAB1 TGF-beta activated kinase 1/MAP3K7 binding protein 1
RELA v-rel avian reticuloendotheliosis viral oncogene homolog A
FADD Fas (TNFRSF6)-associated via death domain
LAMP1 lysosomal-associated membrane protein 1
VPS11 vacuolar protein sorting 11 homolog (S. cerevisiae)
IGF2R insulin-like growth factor 2 receptor
HLA-DQA2 major histocompatibility complex, class II, DQ alpha 2
HLA-DPB1 major histocompatibility complex, class II, DP beta 1
HLA-DPA1 major histocompatibility complex, class II, DP alpha 1
HLA-DOA major histocompatibility complex, class II, DO alpha
CCL2 chemokine (C-C motif) ligand 2
LTBP3 latent transforming growth factor beta binding protein 3
MADCAM1 mucosal vascular addressin cell adhesion molecule 1
FGFR1 fibroblast growth factor receptor 1
CD59 CD59 molecule, complement regulatory protein
CCL4 chemokine (C-C motif) ligand 4
THBS1 thrombospondin 1
IL27 interleukin 27
IFNAR1 interferon (alpha, beta and omega) receptor 1
PDGFRA platelet-derived growth factor receptor, alpha polypeptide
