# Supplementary material for: Molecular characterization of systemic sclerosis esophageal pathology identifies inflammatory and proliferative signatures
Source: Arthritis Res Ther. 2015 Jul 29;17:194. doi: 10.1186/s13075-015-0695-1 (PMC4518531; doi:10.1186/s13075-015-0695-1)
Supplement: Additional file 7: — Gene expression in esophageal biopsies from patients with SSc. (A) Dendrogram of hierarchical clustering of samples based on 3507 probes identified as present in ≥2 arrays with values ≥2-fold change over median. Brackets indicate biopsies from the upper and lower esophagus for an individual that clustered together. SSc patient biopsies clustered independently of lcSSc and dcSSc designation shown in black and red, respectively. An asterisk indicates samples obtained at 6 months. (B) Overview of hierarchically clustered probes. (C) A subset of SSc patients shows overexpression of an inflammatory gene signature (blue and purple clusters). The leaves of the dendrogram indicating the inflammatory subset of arrays are shown in purple. [file 13075_2015_695_MOESM7_ESM.pdf]

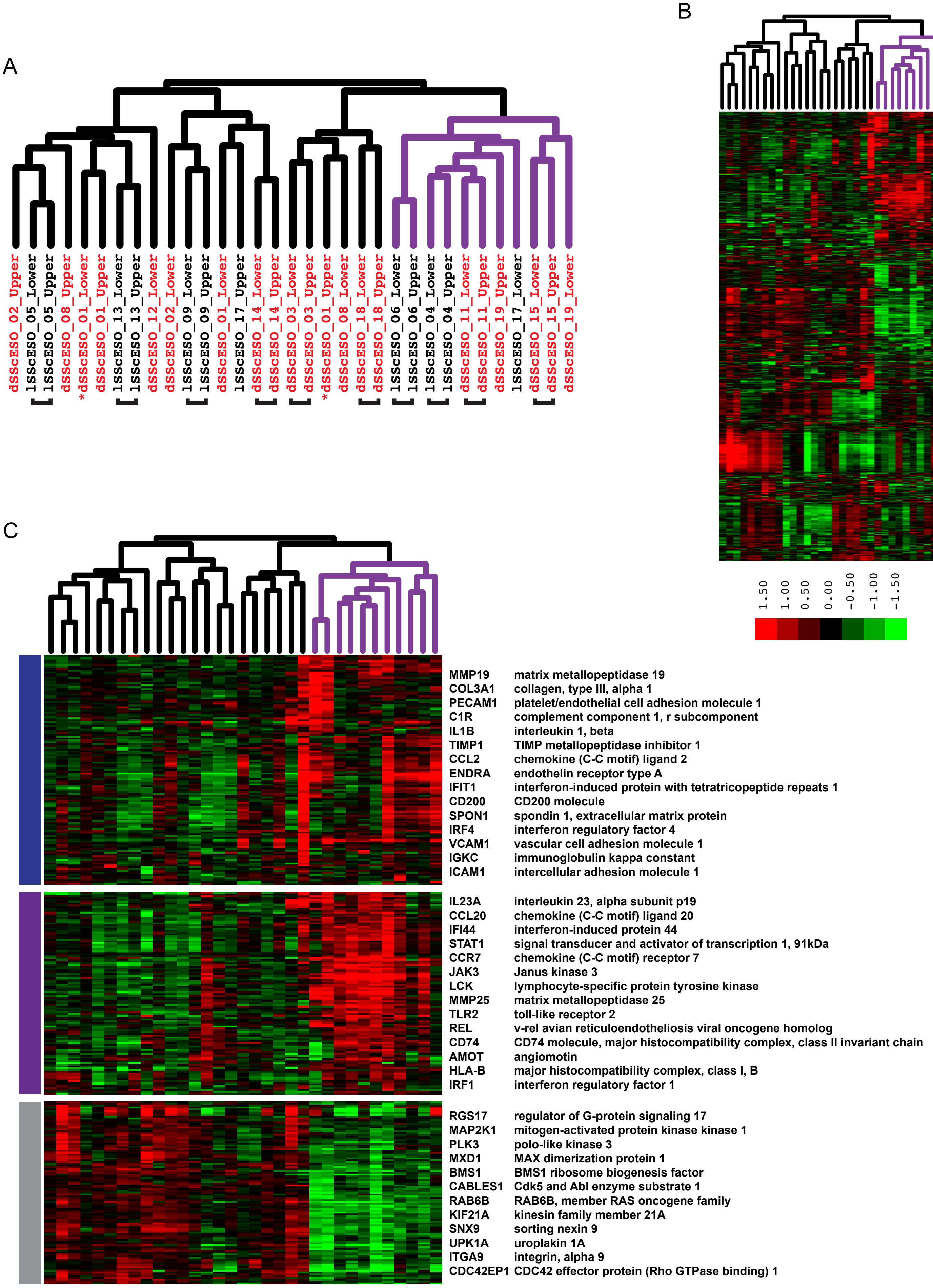
A
dSScESO_02_Upper
lSScESO_05_Lower
lSScESO_05_Upper
dSScESO_08_Upper
*dSScESO_01_Lower
dSScESO_01_Upper
lSScESO_13_Lower
lSScESO_13_Upper
dSScESO_12_Lower
dSScESO_02_Lower
lSScESO_09_Lower
lSScESO_09_Upper
dSScESO_01_Lower
lSScESO_17_Upper
dSScESO_14_Lower
dSScESO_14_Upper
dSScESO_03_Lower
dSScESO_03_Upper
*dSScESO_01_Upper
dSScESO_08_Lower
dSScESO_18_Lower
dSScESO_18_Upper
lSScESO_06_Lower
lSScESO_06_Upper
lSScESO_04_Lower
lSScESO_04_Upper
dSScESO_11_Lower
dSScESO_11_Upper
dSScESO_19_Upper
lSScESO_17_Lower
dSScESO_15_Lower
dSScESO_15_Upper
dSScESO_19_Lower
B
1.50
1.00
0.50
0.00
-0.50
-1.00
-1.50
C
MMP19 matrix metallopeptidase 19
COL3A1 collagen, type III, alpha 1
PECAM1 platelet/endothelial cell adhesion molecule 1
C1R complement component 1, r subcomponent
IL1B interleukin 1, beta
TIMP1 TIMP metallopeptidase inhibitor 1
CCL2 chemokine (C-C motif) ligand 2
ENDRA endothelin receptor type A
IFIT1 interferon-induced protein with tetratricopeptide repeats 1
CD200 CD200 molecule
SPON1 spondin 1, extracellular matrix protein
IRF4 interferon regulatory factor 4
VCAM1 vascular cell adhesion molecule 1
IGKC immunoglobulin kappa constant
ICAM1 intercellular adhesion molecule 1
IL23A interleukin 23, alpha subunit p19
CCL20 chemokine (C-C motif) ligand 20
IFI44 interferon-induced protein 44
STAT1 signal transducer and activator of transcription 1, 91kDa
CCR7 chemokine (C-C motif) receptor 7
JAK3 Janus kinase 3
LCK lymphocyte-specific protein tyrosine kinase
MMP25 matrix metallopeptidase 25
TLR2 toll-like receptor 2
REL v-rel avian reticuloendotheliosis viral oncogene homolog
CD74 CD74 molecule, major histocompatibility complex, class II invariant chain
AMOT angiomotin
HLA-B major histocompatibility complex, class I, B
IRF1 interferon regulatory factor 1
RGS17 regulator of G-protein signaling 17
MAP2K1 mitogen-activated protein kinase kinase 1
PLK3 polo-like kinase 3
MXD1 MAX dimerization protein 1
BMS1 BMS1 ribosome biogenesis factor
CABLES1 Cdk5 and Abl enzyme substrate 1
RAB6B RAB6B, member RAS oncogene family
KIF21A kinesin family member 21A
SNX9 sorting nexin 9
UPK1A uroplakin 1A
ITGA9 integrin, alpha 9
CDC42EP1 CDC42 effector protein (Rho GTPase binding) 1
