# Supplementary material for: Molecular characterization of systemic sclerosis esophageal pathology identifies inflammatory and proliferative signatures
Source: Arthritis Res Ther. 2015 Jul 29;17:194. doi: 10.1186/s13075-015-0695-1 (PMC4518531; doi:10.1186/s13075-015-0695-1)
Supplement: Additional file 12: — Complete g:Profiler results from genes differentially expressed between SSc groups (Fig. 4 ). [file 13075_2015_695_MOESM12_ESM.pdf]

Table S4:

Functional Enrichment Results, Fig 5 Red Cluster

p-value	term ID	term name
2.63E-02	GO:0071495	cellular response to endogenous stimulus
2.20E-04	GO:0070848	response to growth factor stimulus
4.83E-03	GO:0071310	cellular response to organic substance
3.28E-04	GO:0071363	cellular response to growth factor stimulus
1.79E-02	GO:0032870	cellular response to hormone stimulus
2.06E-04	GO:0007167	enzyme linked receptor protein signaling pathway
5.44E-05	GO:0007169	transmembrane receptor protein tyrosine kinase signaling pathway
3.40E-02	GO:0038127	ERBB signaling pathway
2.78E-02	GO:0007173	epidermal growth factor receptor signaling pathway
9.35E-06	GO:0038179	neurotrophin signaling pathway
8.27E-06	GO:0048011	neurotrophin TRK receptor signaling pathway
3.66E-02	GO:0006650	glycerophospholipid metabolic process
1.39E-02	GO:0030518	intracellular steroid hormone receptor signaling pathway
2.89E-02	GO:0044281	small molecule metabolic process
2.52E-02	GO:0016236	macroautophagy
5.89E-03	GO:0038093	Fc receptor signaling pathway
2.74E-04	GO:0043632	modification-dependent macromolecule catabolic process
1.28E-12	GO:0008150	biological_process
4.96E-07	GO:0044699	single-organism process
7.21E-06	GO:0016265	death
8.58E-12	GO:0065007	biological regulation
2.88E-03	GO:0065009	regulation of molecular function
4.86E-02	GO:0044093	positive regulation of molecular function
1.97E-20	GO:0009987	cellular process
1.07E-09	GO:0044763	single-organism cellular process
3.01E-02	GO:0007163	establishment or maintenance of cell polarity
1.16E-03	GO:0007154	cell communication
5.66E-03	GO:0030029	actin filament-based process
3.30E-10	GO:0007049	cell cycle
2.44E-05	GO:0000278	mitotic cell cycle
6.32E-06	GO:0008219	cell death
2.98E-05	GO:0012501	programmed cell death
2.50E-05	GO:0006915	apoptotic process
1.16E-04	GO:0022402	cell cycle process
1.41E-02	GO:0044770	cell cycle phase transition

1.41E-17	GO:0008152	metabolic process
2.36E-21	GO:0044238	primary metabolic process
2.02E-20	GO:0071704	organic substance metabolic process
3.75E-11	GO:1901360	organic cyclic compound metabolic process
4.97E-17	GO:0043170	macromolecule metabolic process
5.92E-08	GO:0010467	gene expression
2.59E-22	GO:0043412	macromolecule modification
2.88E-14	GO:0019538	protein metabolic process
1.74E-20	GO:0036211	protein modification process
1.17E-02	GO:0032259	methylation
1.29E-10	GO:0006807	nitrogen compound metabolic process
2.09E-10	GO:0009058	biosynthetic process
3.52E-11	GO:1901576	organic substance biosynthetic process
1.00E-03	GO:1901362	organic cyclic compound biosynthetic process
9.24E-09	GO:0009059	macromolecule biosynthetic process
1.23E-04	GO:0009056	catabolic process
4.72E-04	GO:1901575	organic substance catabolic process
2.81E-02	GO:0009057	macromolecule catabolic process
3.31E-03	GO:0030163	protein catabolic process
2.39E-22	GO:0044237	cellular metabolic process
4.23E-08	GO:0006793	phosphorus metabolic process
2.22E-08	GO:0006796	phosphate-containing compound metabolic process
2.48E-03	GO:0016310	phosphorylation
4.14E-02	GO:0019637	organophosphate metabolic process
8.69E-23	GO:0044260	cellular macromolecule metabolic process
6.40E-03	GO:0043414	macromolecule methylation
1.06E-19	GO:0044267	cellular protein metabolic process
1.74E-20	GO:0006464	cellular protein modification process
5.04E-09	GO:0070647	protein modification by small protein conjugation or removal
3.01E-07	GO:0032446	protein modification by small protein conjugation
4.23E-07	GO:0016567	protein ubiquitination
5.84E-09	GO:0018193	peptidyl-amino acid modification
3.61E-04	GO:0018196	peptidyl-asparagine modification
1.73E-04	GO:0006468	protein phosphorylation
4.82E-03	GO:0044257	cellular protein catabolic process
1.22E-03	GO:0051603	proteolysis involved in cellular protein catabolic process

2.76E-03	GO:0010498	proteasomal protein catabolic process
4.27E-04	GO:0019941	modification-dependent protein catabolic process
2.61E-04	GO:0006511	ubiquitin-dependent protein catabolic process
4.04E-03	GO:0043161	proteasomal ubiquitin-dependent protein catabolic process
2.15E-04	GO:0044248	cellular catabolic process
1.65E-11	GO:0046483	heterocycle metabolic process
3.13E-10	GO:0006725	cellular aromatic compound metabolic process
1.10E-10	GO:0044249	cellular biosynthetic process
3.86E-04	GO:0018130	heterocycle biosynthetic process
9.75E-04	GO:0019438	aromatic compound biosynthetic process
6.13E-09	GO:0034645	cellular macromolecule biosynthetic process
2.60E-10	GO:0034641	cellular nitrogen compound metabolic process
2.84E-03	GO:0044271	cellular nitrogen compound biosynthetic process
1.34E-11	GO:0006139	nucleobase-containing compound metabolic process
6.14E-09	GO:0090304	nucleic acid metabolic process
5.45E-08	GO:0016070	RNA metabolic process
7.89E-05	GO:0006396	RNA processing
7.60E-04	GO:0034654	nucleobase-containing compound biosynthetic process
4.30E-03	GO:0032774	RNA biosynthetic process
2.81E-04	GO:0006351	transcription, DNA-dependent
4.27E-05	GO:0006366	transcription from RNA polymerase II promoter
1.42E-08	GO:0051179	localization
3.35E-10	GO:0033036	macromolecule localization
9.34E-11	GO:0008104	protein localization
4.69E-10	GO:0051641	cellular localization
4.38E-04	GO:0051640	organelle localization
2.13E-07	GO:0070727	cellular macromolecule localization
5.60E-07	GO:0034613	cellular protein localization
3.46E-08	GO:0071840	cellular component organization or biogenesis
1.96E-07	GO:0016043	cellular component organization
1.13E-04	GO:0061024	membrane organization
3.06E-04	GO:0016044	cellular membrane organization
6.05E-12	GO:0006996	organelle organization
1.61E-04	GO:0007010	cytoskeleton organization
2.71E-02	GO:0030036	actin cytoskeleton organization
3.73E-07	GO:0051234	establishment of localization

1.29E-06	GO:0006810	transport
1.33E-03	GO:0071702	organic substance transport
7.14E-13	GO:0016192	vesicle-mediated transport
2.14E-04	GO:0006897	endocytosis
5.70E-08	GO:0045184	establishment of protein localization
3.52E-08	GO:0015031	protein transport
2.24E-07	GO:0051649	establishment of localization in cell
9.42E-10	GO:0046907	intracellular transport
2.07E-02	GO:0051169	nuclear transport
9.03E-06	GO:0048193	Golgi vesicle transport
3.28E-03	GO:0006890	retrograde vesicle-mediated transport, Golgi to ER
1.15E-05	GO:0006886	intracellular protein transport
1.46E-11	GO:0050789	regulation of biological process
5.76E-08	GO:0048518	positive regulation of biological process
1.54E-02	GO:0044087	regulation of cellular component biogenesis
5.56E-07	GO:0048519	negative regulation of biological process
1.40E-10	GO:0019222	regulation of metabolic process
8.36E-06	GO:0009889	regulation of biosynthetic process
2.99E-10	GO:0080090	regulation of primary metabolic process
8.24E-11	GO:0060255	regulation of macromolecule metabolic process
1.29E-06	GO:0010556	regulation of macromolecule biosynthetic process
9.32E-07	GO:0010468	regulation of gene expression
1.12E-02	GO:0010608	posttranscriptional regulation of gene expression
2.10E-06	GO:0051246	regulation of protein metabolic process
1.52E-03	GO:0009894	regulation of catabolic process
3.46E-09	GO:0009893	positive regulation of metabolic process
8.49E-04	GO:0009891	positive regulation of biosynthetic process
1.18E-09	GO:0010604	positive regulation of macromolecule metabolic process
3.39E-04	GO:0010628	positive regulation of gene expression
5.55E-06	GO:0051247	positive regulation of protein metabolic process
2.92E-04	GO:0010557	positive regulation of macromolecule biosynthetic process
6.16E-04	GO:0009892	negative regulation of metabolic process
1.84E-04	GO:0010605	negative regulation of macromolecule metabolic process
7.54E-04	GO:0010629	negative regulation of gene expression
4.42E-02	GO:0051248	negative regulation of protein metabolic process
7.84E-03	GO:0009890	negative regulation of biosynthetic process

2.10E-03	GO:0010558	negative regulation of macromolecule biosynthetic process
3.41E-03	GO:0050790	regulation of catalytic activity
4.42E-03	GO:0051338	regulation of transferase activity
3.28E-02	GO:0043085	positive regulation of catalytic activity
6.99E-03	GO:0051347	positive regulation of transferase activity
4.83E-07	GO:0051171	regulation of nitrogen compound metabolic process
1.85E-03	GO:0051173	positive regulation of nitrogen compound metabolic process
9.88E-03	GO:0051172	negative regulation of nitrogen compound metabolic process
4.51E-11	GO:0050794	regulation of cellular process
2.92E-08	GO:0048522	positive regulation of cellular process
1.53E-07	GO:0048523	negative regulation of cellular process
1.33E-03	GO:0051726	regulation of cell cycle
4.78E-04	GO:0010646	regulation of cell communication
3.20E-02	GO:0010648	negative regulation of cell communication
3.83E-11	GO:0031323	regulation of cellular metabolic process
4.38E-06	GO:0031326	regulation of cellular biosynthetic process
3.51E-07	GO:2000112	regulation of cellular macromolecule biosynthetic process
7.82E-03	GO:0031329	regulation of cellular catabolic process
1.71E-07	GO:0019219	regulation of nucleobase-containing compound metabolic process
1.61E-05	GO:0051252	regulation of RNA metabolic process
3.35E-05	GO:2001141	regulation of RNA biosynthetic process
1.46E-05	GO:0006355	regulation of transcription, DNA-dependent
5.99E-04	GO:0006357	regulation of transcription from RNA polymerase II promoter
2.58E-02	GO:0051174	regulation of phosphorus metabolic process
2.33E-02	GO:0019220	regulation of phosphate metabolic process
3.99E-02	GO:0042325	regulation of phosphorylation
4.08E-03	GO:0043549	regulation of kinase activity
6.41E-03	GO:0033674	positive regulation of kinase activity
2.61E-09	GO:0031325	positive regulation of cellular metabolic process
1.03E-03	GO:0045935	positive regulation of nucleobase-containing compound metabolic process
3.29E-04	GO:0051254	positive regulation of RNA metabolic process
3.07E-04	GO:0031328	positive regulation of cellular biosynthetic process
4.59E-04	GO:0045893	positive regulation of transcription, DNA-dependent
5.18E-04	GO:0031324	negative regulation of cellular metabolic process
6.26E-03	GO:0031327	negative regulation of cellular biosynthetic process
1.27E-03	GO:2000113	negative regulation of cellular macromolecule biosynthetic process

4.95E-03	GO:0045934	negative regulation of nucleobase-containing compound metabolic process
2.33E-03	GO:0051253	negative regulation of RNA metabolic process
1.36E-03	GO:0045892	negative regulation of transcription, DNA-dependent
3.50E-02	GO:0000122	negative regulation of transcription from RNA polymerase II promoter
2.73E-08	GO:0032268	regulation of cellular protein metabolic process
4.65E-07	GO:0031399	regulation of protein modification process
3.37E-02	GO:0031396	regulation of protein ubiquitination
1.51E-02	GO:0001932	regulation of protein phosphorylation
6.17E-03	GO:0045859	regulation of protein kinase activity
9.04E-06	GO:0032270	positive regulation of cellular protein metabolic process
1.54E-04	GO:0031401	positive regulation of protein modification process
6.30E-05	GO:0051128	regulation of cellular component organization
3.30E-02	GO:0051130	positive regulation of cellular component organization
3.31E-03	GO:0033043	regulation of organelle organization
3.75E-04	GO:0023051	regulation of signaling
4.22E-02	GO:0023057	negative regulation of signaling
2.70E-02	GO:0032989	cellular component morphogenesis
2.92E-02	GO:0032990	cell part morphogenesis
6.20E-03	GO:0000902	cell morphogenesis
1.65E-05	GO:0051716	cellular response to stimulus
8.82E-03	GO:0007165	signal transduction
5.96E-04	GO:0035556	intracellular signal transduction
4.11E-04	GO:0007264	small GTPase mediated signal transduction
1.25E-05	GO:0007265	Ras protein signal transduction
1.75E-03	GO:0007266	Rho protein signal transduction
1.46E-04	GO:0009966	regulation of signal transduction
4.15E-02	GO:0051056	regulation of small GTPase mediated signal transduction
1.56E-02	GO:0046578	regulation of Ras protein signal transduction
2.00E-03	GO:0035023	regulation of Rho protein signal transduction
3.46E-02	GO:0009968	negative regulation of signal transduction
2.75E-05	GO:0033554	cellular response to stress
9.52E-03	GO:0016568	chromatin modification
4.01E-04	GO:0016569	covalent chromatin modification
2.91E-04	GO:0016570	histone modification
3.38E-02	GO:0007015	actin filament organization
2.21E-02	GO:0048858	cell projection morphogenesis

3.43E-02	GO:0022604	regulation of cell morphogenesis
1.49E-02	GO:0048667	cell morphogenesis involved in neuron differentiation
5.54E-03	GO:0006487	protein N-linked glycosylation
1.22E-03	GO:0018279	protein N-linked glycosylation via asparagine
4.28E-03	GO:0048812	neuron projection morphogenesis
4.83E-02	GO:0007409	axonogenesis
3.98E-02	GO:0045860	positive regulation of protein kinase activity
4.27E-03	GO:0044419	interspecies interaction between organisms
4.27E-03	GO:0044403	symbiosis, encompassing mutualism through parasitism
1.08E-03	GO:0016032	viral process
1.60E-02	GO:0030032	lamellipodium assembly
2.17E-02	GO:0071496	cellular response to external stimulus
3.90E-02	GO:0048770	pigment granule
3.90E-02	GO:0042470	melanosome
1.86E-04	GO:0070161	anchoring junction
2.29E-04	GO:0005912	adherens junction
4.99E-02	GO:0005913	cell-cell adherens junction
1.40E-03	GO:0048475	coated membrane
7.12E-12	GO:0032991	macromolecular complex
1.54E-10	GO:0043234	protein complex
9.15E-34	GO:0043226	organelle
2.41E-09	GO:0043228	non-membrane-bounded organelle
4.73E-03	GO:0031982	vesicle
3.33E-02	GO:0031988	membrane-bounded vesicle
6.14E-34	GO:0043227	membrane-bounded organelle
2.60E-16	GO:0031974	membrane-enclosed lumen
3.24E-16	GO:0005623	cell
3.10E-16	GO:0044464	cell part
3.37E-03	GO:0031252	cell leading edge
5.74E-50	GO:0005622	intracellular
2.13E-04	GO:0012505	endomembrane system
1.11E-48	GO:0044424	intracellular part
5.92E-38	GO:0005737	cytoplasm
3.60E-34	GO:0043229	intracellular organelle
2.41E-09	GO:0043232	intracellular non-membrane-bounded organelle
3.50E-04	GO:0005856	cytoskeleton

1.85E-06	GO:0015630	microtubule cytoskeleton
7.90E-34	GO:0043231	intracellular membrane-bounded organelle
1.20E-22	GO:0005634	nucleus
1.55E-22	GO:0044444	cytoplasmic part
1.96E-18	GO:0005829	cytosol
5.22E-03	GO:0048471	perinuclear region of cytoplasm
6.70E-05	GO:0005938	cell cortex
1.81E-03	GO:0044448	cell cortex part
5.03E-03	GO:0030863	cortical cytoskeleton
3.33E-02	GO:0031410	cytoplasmic vesicle
1.03E-04	GO:0005768	endosome
3.07E-09	GO:0005794	Golgi apparatus
1.40E-03	GO:0030117	membrane coat
6.74E-03	GO:0030118	clathrin coat
1.95E-23	GO:0044422	organelle part
6.03E-17	GO:0043233	organelle lumen
5.14E-07	GO:0031090	organelle membrane
1.66E-23	GO:0044446	intracellular organelle part
3.41E-18	GO:0070013	intracellular organelle lumen
1.55E-03	GO:0044431	Golgi apparatus part
7.34E-04	GO:0000139	Golgi membrane
1.93E-18	GO:0044428	nuclear part
5.86E-18	GO:0031981	nuclear lumen
8.70E-15	GO:0005654	nucleoplasm
1.44E-06	GO:0044451	nucleoplasm part
5.63E-04	GO:0005730	nucleolus
2.26E-03	GO:0044430	cytoskeletal part
2.67E-02	GO:0005815	microtubule organizing center
2.12E-03	GO:0005819	spindle
4.47E-02	GO:0030027	lamellipodium
7.59E-04	GO:0003674	molecular_function
1.40E-31	GO:0005488	binding
7.19E-05	GO:1901363	heterocyclic compound binding
7.78E-38	GO:0005515	protein binding
2.27E-04	GO:0008092	cytoskeletal protein binding
8.36E-08	GO:0019899	enzyme binding

2.55E-03	GO:0019900	kinase binding
7.74E-04	GO:0019901	protein kinase binding
8.51E-06	GO:0008134	transcription factor binding
1.22E-02	GO:0051219	phosphoprotein binding
1.46E-03	GO:0045309	protein phosphorylated amino acid binding
1.16E-03	GO:0019904	protein domain specific binding
1.01E-04	GO:0097159	organic cyclic compound binding
7.87E-11	GO:1901265	nucleoside phosphate binding
1.23E-06	GO:0043167	ion binding
1.44E-12	GO:0043168	anion binding
9.91E-07	GO:0035639	purine ribonucleoside triphosphate binding
1.11E-10	GO:0036094	small molecule binding
1.69E-06	GO:0001882	nucleoside binding
1.77E-06	GO:0032549	ribonucleoside binding
1.70E-06	GO:0001883	purine nucleoside binding
1.48E-06	GO:0032550	purine ribonucleoside binding
7.49E-11	GO:0000166	nucleotide binding
6.48E-06	GO:0032553	ribonucleotide binding
2.71E-06	GO:0017076	purine nucleotide binding
3.26E-06	GO:0032555	purine ribonucleotide binding
3.56E-07	GO:0030554	adenyl nucleotide binding
4.72E-07	GO:0032559	adenyl ribonucleotide binding
1.58E-07	GO:0005524	ATP binding
2.08E-02	GO:0003682	chromatin binding
1.30E-05	GO:0008289	lipid binding
1.68E-06	GO:0005543	phospholipid binding
1.07E-03	GO:0035091	phosphatidylinositol binding
3.31E-09	GO:0003824	catalytic activity
8.95E-03	GO:0016874	ligase activity
1.44E-06	GO:0016740	transferase activity
1.00E-05	GO:0016772	transferase activity, transferring phosphorus-containing groups
2.58E-04	GO:0016301	kinase activity
2.86E-05	GO:0016773	phosphotransferase activity, alcohol group as acceptor
2.00E-02	GO:0004672	protein kinase activity
2.31E-07	GO:0000988	protein binding transcription factor activity
2.11E-07	GO:0000989	transcription factor binding transcription factor activity

1.48E-05	GO:0003712	transcription cofactor activity
6.13E-04	GO:0003713	transcription coactivator activity
2.16E-02	GO:0030374	ligand-dependent nuclear receptor transcription coactivator activity
2.66E-03	GO:0008565	protein transporter activity
3.25E-02	GO:0003723	RNA binding
6.45E-45	BIOGRID:0000(	BioGRID interaction data
1.16E-02	CORUM:1413	NCOR1 complex
2.11E-02	HP:0000759	Abnormality of the peripheral nervous system
3.68E-02	KEGG:04530	Tight junction
4.36E-03	KEGG:05212	Pancreatic cancer
1.31E-02	KEGG:05211	Renal cell carcinoma
4.37E-03	KEGG:04360	Axon guidance
2.62E-02	KEGG:05131	Shigellosis
1.28E-03	KEGG:05220	Chronic myeloid leukemia
1.38E-02	KEGG:04722	Neurotrophin signaling pathway
2.47E-02	KEGG:04330	Notch signaling pathway
3.09E-02	KEGG:05219	Bladder cancer
2.82E-02	KEGG:04810	Regulation of actin cytoskeleton
9.74E-03	KEGG:04912	GnRH signaling pathway
3.77E-04	KEGG:04520	Adherens junction
2.76E-03	MI:hsa-miR-497	MI:hsa-miR-497
3.12E-02	MI:mmu-miR-34	MI:mmu-miR-341
3.46E-03	MI:mmu-miR-67	MI:mmu-miR-673-5p
4.17E-02	MI:hsa-miR-197	MI:hsa-miR-197
1.27E-02	MI:mmu-miR-54	MI:mmu-miR-540-5p
2.20E-03	MI:rno-miR-347	MI:rno-miR-347
4.30E-02	MI:mmu-miR-71	MI:mmu-miR-715
3.78E-02	MI:hsa-miR-449	MI:hsa-miR-449b
3.91E-03	MI:mmu-miR-68	MI:mmu-miR-689
2.73E-03	MI:hsa-miR-524	MI:hsa-miR-524-3p
4.72E-05	MI:hsa-miR-874	MI:hsa-miR-874
4.99E-02	MI:hsa-miR-933	MI:hsa-miR-933
1.55E-02	MI:hsa-miR-615	MI:hsa-miR-615-3p
1.77E-02	MI:hsa-miR-490	MI:hsa-miR-490-3p
3.18E-02	MI:mmu-miR-35	MI:mmu-miR-351
5.00E-02	MI:mmu-miR-76	MI:mmu-miR-762

5.59E-04	MI:mmu-miR-70	MI:mmu-miR-702
2.62E-03	MI:hsa-miR-503	MI:hsa-miR-503
2.68E-02	MI:hsa-miR-574	MI:hsa-miR-574-3p
2.89E-03	MI:hsa-miR-214	MI:hsa-miR-214
2.86E-02	MI:hsa-miR-24	MI:hsa-miR-24
1.90E-02	MI:hsa-miR-195	MI:hsa-miR-195
8.04E-05	MI:hsa-miR-92b	MI:hsa-miR-92b*
1.87E-03	MI:hsa-miR-324	MI:hsa-miR-324-5p
3.68E-03	MI:mmu-miR-71	MI:mmu-miR-714
7.86E-07	MI:hsa-miR-324	MI:hsa-miR-324-3p
3.99E-04	MI:hsa-miR-608	MI:hsa-miR-608
1.08E-03	MI:hsa-miR-15b	MI:hsa-miR-15b
1.82E-03	MI:hsa-miR-412	MI:hsa-miR-412
1.87E-03	MI:hsa-miR-483	MI:hsa-miR-483-5p
2.83E-03	MI:hsa-miR-744	MI:hsa-miR-744
1.32E-04	MI:hsa-miR-423	MI:hsa-miR-423-5p
4.13E-05	MI:hsa-miR-296	MI:hsa-miR-296-5p
5.81E-04	MI:hsa-miR-486	MI:hsa-miR-486-3p
1.03E-05	MI:hsa-miR-885	MI:hsa-miR-885-3p
1.26E-04	MI:hsa-miR-615	MI:hsa-miR-615-5p
2.45E-02	MI:hsa-miR-887	MI:hsa-miR-887
1.65E-03	MI:hsa-miR-525	MI:hsa-miR-525-3p
2.64E-04	MI:mmu-miR-70	MI:mmu-miR-705
1.98E-02	MI:mmu-miR-67	MI:mmu-miR-673-3p
3.15E-02	MI:hsa-miR-642	MI:hsa-miR-642
1.06E-02	MI:hsa-miR-602	MI:hsa-miR-602
1.20E-02	REAC:186763	Down-stream signal transduction
1.55E-02	REAC:112412	SOS-mediated signalling
2.87E-02	REAC:77388	SHC-related events
7.76E-03	REAC:112410	SHC-mediated signalling
8.51E-03	REAC:177929	Signaling by EGFR
1.55E-02	REAC:179812	Grb2 events in EGFR signaling
2.87E-02	REAC:180336	Shc events in EGFR signaling
4.67E-06	REAC:166520	Signalling by NGF
4.30E-02	REAC:187037	TRKA signalling from the plasma membrane
3.52E-02	REAC:187687	Signalling to ERKs

1.98E-02	REAC:167044	Signalling to RAS
1.11E-02	REAC:109869	MAP kinase cascade
3.96E-02	REAC:375432	Vamp2 associated Clathrin derived vesicle budding
2.08E-02	REAC:72203	Processing of Capped Intron-Containing Pre-mRNA
1.98E-02	REAC:379716	Cytosolic tRNA aminoacylation
4.77E-02	REAC:72130	Formation of an intermediate Spliceosomal C complex
3.30E-02	REAC:112295	Elongation and Processing of Capped Transcripts
3.30E-02	REAC:76043	Elongation of Intron-Containing Transcripts and co-transcriptional mRNA splicing
3.96E-02	REAC:375434	Vamp8 associated clathrin derived vesicle budding
7.68E-03	TF:M00178_4	Factor: CREB; motif: NSTGACGTMANN; match class: 4
1.48E-02	TF:M00179_4	Factor: ATF2; motif: VGTGACGTMACN; match class: 4
5.29E-03	TF:M00981_2	Factor: CREB, ATF; motif: NTGACGTNA; match class: 2
1.96E-02	TF:M00981_0	Factor: CREB, ATF; motif: NTGACGTNA; match class: 0
1.83E-02	TF:M00965_4	Factor: LXR, PXR, CAR, COUP, RAR; motif: YGAMCTNNASTRACCYN; match class: 4
9.33E-21	TF:M00915_4	Factor: AP-2; motif: SNNNCCNCAGGCN; match class: 4
6.00E-15	TF:M00915_3	Factor: AP-2; motif: SNNNCCNCAGGCN; match class: 3
5.51E-11	TF:M00915_2	Factor: AP-2; motif: SNNNCCNCAGGCN; match class: 2
4.35E-02	TF:M00915_1	Factor: AP-2; motif: SNNNCCNCAGGCN; match class: 1
1.64E-30	TF:M00931_4	Factor: Sp1; motif: GGGGCGGGGC; match class: 4
4.82E-32	TF:M00931_3	Factor: Sp1; motif: GGGGCGGGGC; match class: 3
3.20E-29	TF:M00931_2	Factor: Sp1; motif: GGGGCGGGGC; match class: 2
6.47E-22	TF:M00931_1	Factor: Sp1; motif: GGGGCGGGGC; match class: 1
2.00E-20	TF:M00931_0	Factor: Sp1; motif: GGGGCGGGGC; match class: 0
2.13E-02	TF:M00982_4	Factor: KROX; motif: CCCGCCCCCRCCCC; match class: 4
7.56E-03	TF:M00982_3	Factor: KROX; motif: CCCGCCCCCRCCCC; match class: 3
2.79E-02	TF:M00982_2	Factor: KROX; motif: CCCGCCCCCRCCCC; match class: 2
8.34E-07	TF:M00431_4	Factor: E2F-1; motif: TTTSGCGS; match class: 4
1.25E-06	TF:M00431_3	Factor: E2F-1; motif: TTTSGCGS; match class: 3
2.24E-29	TF:M00932_4	Factor: Sp1; motif: NNGGGGCGGGGNN; match class: 4
1.63E-30	TF:M00932_3	Factor: Sp1; motif: NNGGGGCGGGGNN; match class: 3
7.10E-30	TF:M00932_2	Factor: Sp1; motif: NNGGGGCGGGGNN; match class: 2
4.73E-20	TF:M00932_1	Factor: Sp1; motif: NNGGGGCGGGGNN; match class: 1
5.34E-09	TF:M00932_0	Factor: Sp1; motif: NNGGGGCGGGGNN; match class: 0
4.73E-04	TF:M00444_4	Factor: VDR; motif: GGGKNARNRRGGWSA; match class: 4
1.27E-03	TF:M00444_3	Factor: VDR; motif: GGGKNARNRRGGWSA; match class: 3
6.77E-31	TF:M00196_4	Factor: Sp1; motif: NGGGGGCGGGGYN; match class: 4

1.10E-29	TF:M00196_3	Factor: Sp1; motif: NGGGGGCGGGGYN; match class: 3
2.53E-26	TF:M00196_2	Factor: Sp1; motif: NGGGGGCGGGGYN; match class: 2
2.27E-17	TF:M00196_1	Factor: Sp1; motif: NGGGGGCGGGGYN; match class: 1
1.44E-08	TF:M00196_0	Factor: Sp1; motif: NGGGGGCGGGGYN; match class: 0
7.73E-04	TF:M00469_4	Factor: AP-2alpha; motif: GCCNNNRGS; match class: 4
7.73E-04	TF:M00469_0	Factor: AP-2alpha; motif: GCCNNNRGS; match class: 0
4.14E-22	TF:M00933_4	Factor: Sp1; motif: CCCCGCCCCN; match class: 4
7.91E-22	TF:M00933_3	Factor: Sp1; motif: CCCCGCCCCN; match class: 3
2.40E-21	TF:M00933_2	Factor: Sp1; motif: CCCCGCCCCN; match class: 2
1.30E-20	TF:M00933_1	Factor: Sp1; motif: CCCCGCCCCN; match class: 1
2.94E-08	TF:M00933_0	Factor: Sp1; motif: CCCCGCCCCN; match class: 0
1.52E-04	TF:M01035_0	Factor: YY1; motif: NYNKCCATNTT; match class: 0
1.01E-33	TF:M00008_4	Factor: Sp1; motif: GGGGCGGGGT; match class: 4
6.38E-33	TF:M00008_3	Factor: Sp1; motif: GGGGCGGGGT; match class: 3
4.63E-21	TF:M00008_2	Factor: Sp1; motif: GGGGCGGGGT; match class: 2
2.89E-02	TF:M00940_4	Factor: E2F-1; motif: NTTTCGCGCS; match class: 4
9.72E-14	TF:M00189_4	Factor: AP-2; motif: MKCCCSCNGGCG; match class: 4
1.33E-11	TF:M00189_3	Factor: AP-2; motif: MKCCCSCNGGCG; match class: 3
3.35E-03	TF:M00189_2	Factor: AP-2; motif: MKCCCSCNGGCG; match class: 2
2.38E-15	TF:M00695_0	Factor: ETF; motif: GVGGMGG; match class: 0
1.07E-16	TF:M00025_4	Factor: Elk-1; motif: NNNNCCGGAARTNN; match class: 4
2.72E-14	TF:M00025_3	Factor: Elk-1; motif: NNNNCCGGAARTNN; match class: 3
1.39E-09	TF:M00025_2	Factor: Elk-1; motif: NNNNCCGGAARTNN; match class: 2
2.26E-05	TF:M00025_1	Factor: Elk-1; motif: NNNNCCGGAARTNN; match class: 1
8.26E-03	TF:M00470_4	Factor: AP-2gamma; motif: GCCYNNGGS; match class: 4
8.26E-03	TF:M00470_2	Factor: AP-2gamma; motif: GCCYNNGGS; match class: 2
1.21E-04	TF:M01072_4	Factor: HIC1; motif: NSNNNNTGCCCSSNN; match class: 4
7.66E-03	TF:M01072_3	Factor: HIC1; motif: NSNNNNTGCCCSSNN; match class: 3
4.88E-16	TF:M00800_4	Factor: AP-2; motif: GSCCSCRGGCNRNRNN; match class: 4
9.31E-11	TF:M00800_3	Factor: AP-2; motif: GSCCSCRGGCNRNRNN; match class: 3
5.62E-07	TF:M00800_2	Factor: AP-2; motif: GSCCSCRGGCNRNRNN; match class: 2
8.32E-41	TF:M00803_0	Factor: E2F; motif: GGCGSG; match class: 0
5.28E-03	TF:M00427_4	Factor: E2F; motif: TTTSGCGS; match class: 4
1.06E-04	TF:M01035_2	Factor: YY1; motif: NYNKCCATNTT; match class: 2
3.87E-12	TF:M01100_4	Factor: LRF; motif: VNNRMCCCC; match class: 4
1.35E-15	TF:M01100_3	Factor: LRF; motif: VNNRMCCCC; match class: 3

2.14E-14	TF:M01100_2	Factor: LRF; motif: VNNRMCCCC; match class: 2
1.59E-05	TF:M01100_1	Factor: LRF; motif: VNNRMCCCC; match class: 1
1.50E-21	TF:M00428_4	Factor: E2F-1; motif: NKTSSCGC; match class: 4
2.89E-19	TF:M00428_3	Factor: E2F-1; motif: NKTSSCGC; match class: 3
3.09E-08	TF:M00428_2	Factor: E2F-1; motif: NKTSSCGC; match class: 2
6.25E-03	TF:M00428_1	Factor: E2F-1; motif: NKTSSCGC; match class: 1

Functional Enrichment Results, Fig 5 Purple Cluster

p-value	term ID	term name
4.24E-03	GO:0006793	phosphorus metabolic process
8.51E-03	GO:0006796	phosphate-containing compound metabolic process
1.06E-02	GO:0016310	phosphorylation
6.30E-04	GO:0001816	cytokine production
2.47E-02	GO:0001817	regulation of cytokine production
4.45E-02	GO:0043551	regulation of phosphatidylinositol 3-kinase activity
1.03E-02	GO:0071887	leukocyte apoptotic process
3.22E-03	GO:0050817	coagulation
3.61E-06	GO:0008150	biological_process
1.94E-11	GO:0050896	response to stimulus
1.26E-02	GO:0042221	response to chemical stimulus
3.16E-05	GO:0010033	response to organic substance
1.02E-09	GO:0006950	response to stress
4.21E-07	GO:0006952	defense response
3.23E-12	GO:0009611	response to wounding
1.22E-04	GO:0042060	wound healing
2.96E-05	GO:0006954	inflammatory response
2.27E-06	GO:0009607	response to biotic stimulus
2.56E-07	GO:0051707	response to other organism
2.00E-03	GO:0009615	response to virus
2.13E-02	GO:0002237	response to molecule of bacterial origin
3.35E-04	GO:0040011	locomotion
1.83E-12	GO:0044699	single-organism process
1.22E-07	GO:0016265	death
8.10E-09	GO:0065007	biological regulation
4.97E-04	GO:0065008	regulation of biological quality
2.66E-02	GO:0050878	regulation of body fluid levels
3.56E-03	GO:0007599	hemostasis
2.61E-03	GO:0007596	blood coagulation
1.00E-07	GO:0065009	regulation of molecular function
5.12E-09	GO:0044093	positive regulation of molecular function
8.39E-07	GO:0009987	cellular process
1.50E-10	GO:0044763	single-organism cellular process
5.09E-03	GO:0006928	cellular component movement
4.15E-07	GO:0007154	cell communication

6.81E-10	GO:0001775	cell activation
4.14E-08	GO:0051716	cellular response to stimulus
1.09E-07	GO:0008219	cell death
5.79E-08	GO:0012501	programmed cell death
1.30E-07	GO:0006915	apoptotic process
2.08E-02	GO:0032502	developmental process
5.98E-06	GO:0051179	localization
8.86E-05	GO:0051674	localization of cell
8.86E-05	GO:0048870	cell motility
1.23E-05	GO:0016477	cell migration
1.12E-16	GO:0002376	immune system process
5.39E-05	GO:0002252	immune effector process
2.40E-11	GO:0006955	immune response
3.22E-02	GO:0002250	adaptive immune response
3.49E-02	GO:0045087	innate immune response
1.37E-07	GO:0045321	leukocyte activation
3.29E-02	GO:0002274	myeloid leukocyte activation
4.53E-07	GO:0046649	lymphocyte activation
3.06E-08	GO:0042110	T cell activation
7.33E-06	GO:0050900	leukocyte migration
5.10E-07	GO:0022610	biological adhesion
1.04E-06	GO:0007155	cell adhesion
7.26E-07	GO:0023052	signaling
7.26E-07	GO:0044700	single organism signaling
2.37E-07	GO:0050789	regulation of biological process
3.43E-10	GO:0048518	positive regulation of biological process
1.94E-09	GO:0048583	regulation of response to stimulus
1.76E-05	GO:0048584	positive regulation of response to stimulus
1.04E-02	GO:0080134	regulation of response to stress
2.47E-02	GO:0048519	negative regulation of biological process
4.90E-05	GO:0050794	regulation of cellular process
1.47E-06	GO:0048522	positive regulation of cellular process
3.01E-04	GO:0010646	regulation of cell communication
3.77E-02	GO:0010647	positive regulation of cell communication
9.10E-06	GO:0050865	regulation of cell activation
1.55E-05	GO:0050867	positive regulation of cell activation

1.97E-06	GO:0010941	regulation of cell death
7.06E-07	GO:0043067	regulation of programmed cell death
8.85E-07	GO:0042981	regulation of apoptotic process
2.50E-02	GO:2000106	regulation of leukocyte apoptotic process
5.47E-08	GO:0007165	signal transduction
9.11E-08	GO:0035556	intracellular signal transduction
4.60E-02	GO:0097190	apoptotic signaling pathway
2.98E-07	GO:0002682	regulation of immune system process
7.54E-05	GO:0002694	regulation of leukocyte activation
6.43E-05	GO:0051249	regulation of lymphocyte activation
6.10E-05	GO:0050863	regulation of T cell activation
6.19E-08	GO:0002684	positive regulation of immune system process
6.62E-05	GO:0002696	positive regulation of leukocyte activation
4.08E-04	GO:0051251	positive regulation of lymphocyte activation
1.61E-03	GO:0050870	positive regulation of T cell activation
1.39E-05	GO:0050776	regulation of immune response
9.35E-05	GO:0050778	positive regulation of immune response
5.20E-05	GO:0002253	activation of immune response
2.51E-04	GO:0023051	regulation of signaling
3.53E-02	GO:0023056	positive regulation of signaling
6.92E-05	GO:0009966	regulation of signal transduction
2.34E-02	GO:0009967	positive regulation of signal transduction
1.08E-05	GO:0050790	regulation of catalytic activity
7.58E-03	GO:0051336	regulation of hydrolase activity
9.95E-07	GO:0043085	positive regulation of catalytic activity
6.74E-05	GO:0051345	positive regulation of hydrolase activity
1.87E-02	GO:0048513	organ development
1.76E-04	GO:0002520	immune system development
1.41E-04	GO:0048534	hematopoietic or lymphoid organ development
7.18E-04	GO:0030097	hemopoiesis
2.59E-04	GO:0002521	leukocyte differentiation
2.24E-02	GO:1902107	positive regulation of leukocyte differentiation
1.55E-05	GO:0030098	lymphocyte differentiation
2.61E-06	GO:0030217	T cell differentiation
2.47E-02	GO:0051270	regulation of cellular component movement
1.40E-02	GO:2000145	regulation of cell motility

9.60E-03	GO:0030334	regulation of cell migration
1.18E-02	GO:0060326	cell chemotaxis
3.29E-02	GO:0030595	leukocyte chemotaxis
4.54E-02	GO:0071621	granulocyte chemotaxis
5.64E-03	GO:0045619	regulation of lymphocyte differentiation
3.12E-03	GO:0045580	regulation of T cell differentiation
1.55E-03	GO:0045621	positive regulation of lymphocyte differentiation
3.35E-02	GO:0045582	positive regulation of T cell differentiation
3.12E-02	GO:0005623	cell
3.07E-02	GO:0044464	cell part
1.89E-03	GO:0005622	intracellular
1.34E-03	GO:0044424	intracellular part
3.45E-13	GO:0005737	cytoplasm
1.62E-07	GO:0044444	cytoplasmic part
2.50E-03	GO:0005829	cytosol
1.81E-03	GO:0044459	plasma membrane part
7.53E-03	GO:0015629	actin cytoskeleton
7.63E-03	GO:0030055	cell-substrate junction
4.77E-02	GO:0005912	adherens junction
1.36E-02	GO:0005924	cell-substrate adherens junction
2.97E-02	GO:0005925	focal adhesion
2.02E-02	GO:0005887	integral to plasma membrane
5.44E-06	GO:0005488	binding
3.25E-10	GO:0005515	protein binding
5.99E-03	GO:0004896	cytokine receptor activity
2.11E-16	BIOGRID:00000	BioGRID interaction data
4.98E-02	CORUM:575	ABIN2-NFKB1-MAP3K8 complex
1.16E-02	KEGG:05162	Measles
7.07E-03	KEGG:04060	Cytokine-cytokine receptor interaction
1.78E-02	KEGG:04062	Chemokine signaling pathway
3.88E-02	KEGG:04064	NF-kappa B signaling pathway
6.36E-04	KEGG:04668	TNF signaling pathway
1.08E-02	KEGG:04621	NOD-like receptor signaling pathway
2.14E-03	KEGG:05202	Transcriptional misregulation in cancer
7.23E-03	KEGG:04660	T cell receptor signaling pathway
3.22E-02	KEGG:05142	Chagas disease (American trypanosomiasis)

1.71E-03	REAC:109582	Hemostasis
1.07E-02	REAC:202733	Cell surface interactions at the vascular wall
3.71E-02	REAC:182594	Association of INK4A with Cdk4/6
2.28E-03	REAC:380108	Chemokine receptors bind chemokines
1.51E-03	TF:M00052_4	Factor: NF-kappaB (p65); motif: GGGRATTTCC; match class: 4
9.41E-03	TF:M00054_3	Factor: NF-kappaB; motif: GGGAMTTYCC; match class: 3
6.69E-03	TF:M00054_2	Factor: NF-kappaB; motif: GGGAMTTYCC; match class: 2
4.66E-02	TF:M00194_4	Factor: NF-kappaB; motif: NGGGGAMTTTCCNN; match class: 4
3.25E-03	TF:M00774_1	Factor: NF-kappaB; motif: NNNNKGGRAANTCCCN; match class: 1
8.70E-03	TF:M00774_0	Factor: NF-kappaB; motif: NNNNKGGRAANTCCCN; match class: 0

Functional Enrichment Results, Fig 5 Brown Cluster

p-value	term ID	term name
1.79E-03	GO:0007154	cell communication
4.69E-02	GO:0008150	biological_process
3.11E-03	GO:0065007	biological regulation
3.06E-03	GO:0032502	developmental process
1.01E-03	GO:0048856	anatomical structure development
1.40E-02	GO:0050789	regulation of biological process
1.07E-02	GO:0050794	regulation of cellular process
1.58E-02	GO:0007275	multicellular organismal development
4.10E-02	GO:0048102	autophagic cell death
2.03E-03	GO:0005911	cell-cell junction
9.58E-03	GO:0030057	desmosome
6.26E-03	GO:0001533	cornified envelope
2.67E-03	GO:0005622	intracellular
5.31E-03	GO:0044424	intracellular part
1.45E-07	GO:0005737	cytoplasm
2.20E-02	GO:0005515	protein binding
1.70E-02	GO:0008289	lipid binding
4.23E-02	MI:hsa-miR-214	MI:hsa-miR-214
5.51E-05	TF:M00931_4	Factor: Sp1; motif: GGGGCGGGGC; match class: 4
4.73E-05	TF:M00931_3	Factor: Sp1; motif: GGGGCGGGGC; match class: 3
8.77E-04	TF:M00931_2	Factor: Sp1; motif: GGGGCGGGGC; match class: 2
5.28E-03	TF:M00931_1	Factor: Sp1; motif: GGGGCGGGGC; match class: 1
7.23E-03	TF:M00931_0	Factor: Sp1; motif: GGGGCGGGGC; match class: 0
1.67E-06	TF:M00932_4	Factor: Sp1; motif: NNGGGGCGGGGNN; match class: 4
1.27E-04	TF:M00932_3	Factor: Sp1; motif: NNGGGGCGGGGNN; match class: 3
1.75E-03	TF:M00932_2	Factor: Sp1; motif: NNGGGGCGGGGNN; match class: 2
1.07E-04	TF:M00932_1	Factor: Sp1; motif: NNGGGGCGGGGNN; match class: 1
4.70E-02	TF:M00982_4	Factor: KROX; motif: CCCGCCCCCRCCCC; match class: 4
2.54E-02	TF:M00469_4	Factor: AP-2alpha; motif: GCCNNNRGS; match class: 4
2.54E-02	TF:M00469_0	Factor: AP-2alpha; motif: GCCNNNRGS; match class: 0
7.05E-05	TF:M00196_4	Factor: Sp1; motif: NGGGGGCGGGGYN; match class: 4
2.12E-05	TF:M00196_3	Factor: Sp1; motif: NGGGGGCGGGGYN; match class: 3
1.30E-05	TF:M00196_2	Factor: Sp1; motif: NGGGGGCGGGGYN; match class: 2
6.34E-03	TF:M00196_1	Factor: Sp1; motif: NGGGGGCGGGGYN; match class: 1
6.26E-03	TF:M01073_3	Factor: HIC1; motif: NNNGGKTGCCCSNNNNNN; match class: 3

6.34E-04	TF:M00189_4	Factor: AP-2; motif: MKCCCSCNGGCG; match class: 4
1.24E-02	TF:M00915_4	Factor: AP-2; motif: SNNNCCNCAGGCN; match class: 4
3.21E-03	TF:M00649_0	Factor: MAZ; motif: GGGGAGGG; match class: 0
3.72E-02	TF:M01100_2	Factor: LRF; motif: VNNRMCCCC; match class: 2
3.07E-02	TF:M01100_1	Factor: LRF; motif: VNNRMCCCC; match class: 1
1.85E-04	TF:M01100_0	Factor: LRF; motif: VNNRMCCCC; match class: 0
1.39E-05	TF:M00933_4	Factor: Sp1; motif: CCCCGCCCCN; match class: 4
5.31E-05	TF:M00933_3	Factor: Sp1; motif: CCCCGCCCCN; match class: 3
5.71E-03	TF:M00933_2	Factor: Sp1; motif: CCCCGCCCCN; match class: 2
2.05E-03	TF:M00933_1	Factor: Sp1; motif: CCCCGCCCCN; match class: 1
5.52E-03	TF:M00695_0	Factor: ETF; motif: GVGGMGG; match class: 0
5.56E-03	TF:M00800_4	Factor: AP-2; motif: GSCCSCRGGCNRNRNN; match class: 4
1.11E-03	TF:M00800_3	Factor: AP-2; motif: GSCCSCRGGCNRNRNN; match class: 3
3.49E-04	TF:M00008_4	Factor: Sp1; motif: GGGGCGGGGT; match class: 4
8.99E-04	TF:M00008_3	Factor: Sp1; motif: GGGGCGGGGT; match class: 3
1.48E-02	TF:M00008_2	Factor: Sp1; motif: GGGGCGGGGT; match class: 2

Functional Enrichment Results, Fig 5 Black Cluster

p-value	term ID	term name
2.06E-02	GO:0006605	protein targeting
2.31E-02	GO:0006612	protein targeting to membrane
3.81E-02	GO:0010171	body morphogenesis
4.25E-02	GO:0060323	head morphogenesis
3.55E-02	GO:0048869	cellular developmental process
4.64E-02	GO:0046907	intracellular transport
1.47E-02	GO:0060271	cilium morphogenesis
2.54E-02	GO:0042384	cilium assembly
3.91E-02	GO:0000280	nuclear division
3.91E-02	GO:0007067	mitosis
8.44E-03	GO:0070727	cellular macromolecule localization
7.12E-03	GO:0034613	cellular protein localization
4.32E-02	GO:0033365	protein localization to organelle
9.61E-03	GO:0072594	establishment of protein localization to organelle
8.46E-03	GO:0030219	megakaryocyte differentiation
7.68E-03	GO:0045652	regulation of megakaryocyte differentiation
1.89E-03	GO:0045653	negative regulation of megakaryocyte differentiation
5.32E-11	GO:0008152	metabolic process
2.39E-06	GO:0044238	primary metabolic process
3.66E-07	GO:0071704	organic substance metabolic process
1.21E-09	GO:1901360	organic cyclic compound metabolic process
6.10E-09	GO:0043170	macromolecule metabolic process
5.49E-11	GO:0010467	gene expression
3.65E-06	GO:0009058	biosynthetic process
2.11E-06	GO:1901576	organic substance biosynthetic process
4.25E-03	GO:1901362	organic cyclic compound biosynthetic process
2.53E-06	GO:0009059	macromolecule biosynthetic process
5.52E-09	GO:0006807	nitrogen compound metabolic process
5.59E-08	GO:0009987	cellular process
1.03E-13	GO:0044237	cellular metabolic process
1.44E-12	GO:0044260	cellular macromolecule metabolic process
5.59E-11	GO:0046483	heterocycle metabolic process
2.61E-11	GO:0006725	cellular aromatic compound metabolic process
1.49E-02	GO:0006091	generation of precursor metabolites and energy
6.72E-06	GO:0006119	oxidative phosphorylation

1.22E-05	GO:0044249	cellular biosynthetic process
1.05E-03	GO:0018130	heterocycle biosynthetic process
6.70E-04	GO:0019438	aromatic compound biosynthetic process
4.06E-06	GO:0034645	cellular macromolecule biosynthetic process
2.78E-08	GO:0034641	cellular nitrogen compound metabolic process
4.57E-03	GO:0044271	cellular nitrogen compound biosynthetic process
2.24E-11	GO:0006139	nucleobase-containing compound metabolic process
4.92E-11	GO:0090304	nucleic acid metabolic process
6.34E-08	GO:0016070	RNA metabolic process
2.87E-02	GO:0016071	mRNA metabolic process
6.63E-05	GO:0006396	RNA processing
3.79E-02	GO:0006259	DNA metabolic process
2.49E-04	GO:0071103	DNA conformation change
8.26E-04	GO:0034654	nucleobase-containing compound biosynthetic process
1.53E-03	GO:0032774	RNA biosynthetic process
4.02E-02	GO:0006351	transcription, DNA-dependent
3.01E-02	GO:0019083	viral transcription
2.15E-11	GO:0071840	cellular component organization or biogenesis
7.57E-05	GO:0044085	cellular component biogenesis
4.69E-10	GO:0016043	cellular component organization
2.62E-02	GO:0043933	macromolecular complex subunit organization
1.18E-06	GO:0071824	protein-DNA complex subunit organization
1.36E-02	GO:0022607	cellular component assembly
2.18E-09	GO:0006996	organelle organization
2.87E-05	GO:0051276	chromosome organization
1.23E-02	GO:0032200	telomere organization
1.05E-02	GO:0000723	telomere maintenance
1.73E-04	GO:0006325	chromatin organization
1.38E-04	GO:0034728	nucleosome organization
1.90E-04	GO:0006333	chromatin assembly or disassembly
1.38E-04	GO:0034724	DNA replication-independent nucleosome organization
3.63E-02	GO:0016568	chromatin modification
4.41E-03	GO:0006338	chromatin remodeling
2.61E-04	GO:0031055	chromatin remodeling at centromere
6.45E-04	GO:0043044	ATP-dependent chromatin remodeling
8.36E-04	GO:0043486	histone exchange

9.27E-04	GO:0006323	DNA packaging
5.52E-05	GO:0031497	chromatin assembly
3.20E-04	GO:0034622	cellular macromolecular complex assembly
2.62E-07	GO:0065004	protein-DNA complex assembly
3.87E-04	GO:0034508	centromere complex assembly
3.57E-05	GO:0006334	nucleosome assembly
1.38E-04	GO:0006336	DNA replication-independent nucleosome assembly
1.38E-04	GO:0034080	CENP-A containing nucleosome assembly at centromere
2.93E-02	GO:0048519	negative regulation of biological process
3.76E-02	GO:0048523	negative regulation of cellular process
1.35E-02	GO:0019222	regulation of metabolic process
5.24E-03	GO:0060255	regulation of macromolecule metabolic process
3.43E-04	GO:0010468	regulation of gene expression
5.04E-03	GO:0009889	regulation of biosynthetic process
2.33E-03	GO:0010556	regulation of macromolecule biosynthetic process
4.25E-02	GO:0080090	regulation of primary metabolic process
1.50E-02	GO:0051171	regulation of nitrogen compound metabolic process
1.12E-03	GO:0009892	negative regulation of metabolic process
1.21E-03	GO:0051172	negative regulation of nitrogen compound metabolic process
3.34E-04	GO:0010605	negative regulation of macromolecule metabolic process
8.80E-03	GO:0010629	negative regulation of gene expression
5.54E-04	GO:0009890	negative regulation of biosynthetic process
1.24E-04	GO:0010558	negative regulation of macromolecule biosynthetic process
3.13E-02	GO:0031323	regulation of cellular metabolic process
8.68E-03	GO:0031326	regulation of cellular biosynthetic process
3.76E-03	GO:2000112	regulation of cellular macromolecule biosynthetic process
5.65E-03	GO:0019219	regulation of nucleobase-containing compound metabolic process
2.34E-02	GO:0051252	regulation of RNA metabolic process
4.77E-02	GO:0051254	positive regulation of RNA metabolic process
8.67E-03	GO:0031324	negative regulation of cellular metabolic process
5.78E-04	GO:0031327	negative regulation of cellular biosynthetic process
4.38E-04	GO:2000113	negative regulation of cellular macromolecule biosynthetic process
1.31E-03	GO:0045934	negative regulation of nucleobase-containing compound metabolic process
2.63E-03	GO:0051253	negative regulation of RNA metabolic process
4.12E-07	GO:0022900	electron transport chain
3.75E-04	GO:0015980	energy derivation by oxidation of organic compounds

4.15E-08	GO:0045333	cellular respiration
2.89E-10	GO:0022904	respiratory electron transport chain
9.34E-06	GO:0042773	ATP synthesis coupled electron transport
9.34E-06	GO:0042775	mitochondrial ATP synthesis coupled electron transport
7.69E-06	GO:0006120	mitochondrial electron transport, NADH to ubiquinone
1.78E-02	GO:0044267	cellular protein metabolic process
1.11E-02	GO:0006412	translation
5.82E-21	GO:0043226	organelle
1.75E-10	GO:0043228	non-membrane-bounded organelle
2.60E-17	GO:0043227	membrane-bounded organelle
9.68E-09	GO:0032991	macromolecular complex
6.87E-04	GO:0032993	protein-DNA complex
1.14E-11	GO:0031974	membrane-enclosed lumen
7.08E-04	GO:0005623	cell
6.94E-04	GO:0044464	cell part
1.98E-04	GO:0031975	envelope
1.44E-20	GO:0005622	intracellular
8.39E-22	GO:0044424	intracellular part
5.39E-10	GO:0005737	cytoplasm
2.37E-04	GO:0030529	ribonucleoprotein complex
2.13E-21	GO:0043229	intracellular organelle
1.75E-10	GO:0043232	intracellular non-membrane-bounded organelle
3.64E-03	GO:0005694	chromosome
1.79E-17	GO:0043231	intracellular membrane-bounded organelle
1.55E-10	GO:0005634	nucleus
1.69E-04	GO:0044444	cytoplasmic part
5.99E-05	GO:0005739	mitochondrion
8.91E-03	GO:0005840	ribosome
1.54E-15	GO:0044422	organelle part
1.23E-10	GO:0043233	organelle lumen
3.37E-16	GO:0044446	intracellular organelle part
2.74E-11	GO:0070013	intracellular organelle lumen
1.59E-04	GO:0031967	organelle envelope
1.06E-03	GO:0019866	organelle inner membrane
2.09E-09	GO:0044428	nuclear part
5.07E-11	GO:0031981	nuclear lumen

1.37E-08	GO:0005654	nucleoplasm
2.72E-03	GO:0044451	nucleoplasm part
1.02E-03	GO:0005730	nucleolus
1.08E-06	GO:0044429	mitochondrial part
3.93E-05	GO:0005740	mitochondrial envelope
1.01E-05	GO:0030964	NADH dehydrogenase complex
3.76E-05	GO:0070469	respiratory chain
1.01E-05	GO:0045271	respiratory chain complex I
4.58E-05	GO:0000786	nucleosome
3.65E-05	GO:0031966	mitochondrial membrane
7.35E-06	GO:0044455	mitochondrial membrane part
1.27E-03	GO:0005743	mitochondrial inner membrane
2.29E-05	GO:0005746	mitochondrial respiratory chain
1.01E-05	GO:0005747	mitochondrial respiratory chain complex I
2.54E-02	GO:1901363	heterocyclic compound binding
4.21E-02	GO:0097159	organic cyclic compound binding
1.13E-04	GO:0003676	nucleic acid binding
8.79E-04	GO:0003723	RNA binding
1.14E-02	GO:0005515	protein binding
3.45E-02	GO:0003712	transcription cofactor activity
4.38E-02	GO:0000988	protein binding transcription factor activity
2.27E-02	GO:0016651	oxidoreductase activity, acting on NAD(P)H
8.68E-07	GO:0003954	NADH dehydrogenase activity
7.86E-05	GO:0016655	oxidoreductase activity, acting on NAD(P)H, quinone or similar compound as acceptor
8.68E-07	GO:0050136	NADH dehydrogenase (quinone) activity
8.68E-07	GO:0008137	NADH dehydrogenase (ubiquinone) activity
2.36E-20	BIOGRID:00000	BioGRID interaction data
9.81E-03	CORUM:3137	MASH1 promoter-coactivator complex
3.93E-03	CORUM:1227	H2AX complex II
1.28E-04	CORUM:178	Respiratory chain complex I (holoenzyme), mitochondrial
1.33E-02	CORUM:1149	Histone H3.1 complex
3.16E-02	CORUM:1223	H2AX complex, isolated from cells without IR exposure
5.13E-06	CORUM:2914	Respiratory chain complex I (beta subunit) mitochondrial
4.07E-02	CORUM:1150	Histone H3.3 complex
5.58E-03	HP:0002921	Abnormality of the cerebrospinal fluid
2.37E-04	HP:0002490	Increased CSF lactate

1.55E-02	HP:0002553	Highly arched eyebrow
1.43E-03	HP:0001427	Mitochondrial inheritance
3.08E-03	KEGG:03010	Ribosome
2.51E-07	KEGG:05012	Parkinson's disease
6.17E-06	KEGG:05010	Alzheimer's disease
2.68E-02	KEGG:05203	Viral carcinogenesis
1.88E-07	KEGG:00190	Oxidative phosphorylation
1.06E-08	KEGG:05016	Huntington's disease
1.46E-03	KEGG:05034	Alcoholism
1.60E-03	MI:hsa-miR-590-3p	MI:hsa-miR-590-3p
6.88E-04	REAC:157579	Telomere Maintenance
3.77E-03	REAC:171306	Packaging Of Telomere Ends
3.77E-03	REAC:176700	Incorporation Of Extended And Processed Telomere End Into Higher Order T-Loop And Associated Protein Structure
3.77E-03	REAC:181450	Incorporation Of Extended And Processed Telomere End Into Associated Protein Structure
1.24E-04	REAC:163685	Integration of energy metabolism
6.89E-05	REAC:381150	Diabetes pathways
6.99E-06	REAC:265764	Glucose Regulation of Insulin Secretion
1.00E-07	REAC:163200	Electron Transport Chain
4.93E-06	REAC:163217	NADH enters the respiratory chain at Complex I
1.96E-02	TF:M00428_4	Factor: E2F-1; motif: NKTSSCGC; match class: 4
2.38E-02	TF:M01035_0	Factor: YY1; motif: NYNKCCATNTT; match class: 0
1.39E-02	TF:M00803_0	Factor: E2F; motif: GGCGSG; match class: 0
