# Supplementary material for: Molecular characterization of systemic sclerosis esophageal pathology identifies inflammatory and proliferative signatures
Source: Arthritis Res Ther. 2015 Jul 29;17:194. doi: 10.1186/s13075-015-0695-1 (PMC4518531; doi:10.1186/s13075-015-0695-1)
Supplement: Additional file 14: — Esophageal histological scoring. [file 13075_2015_695_MOESM14_ESM.pdf]

Table S3: Esophageal histological scoring and endoscopy findings

Study Code	Age**/ Sex	Date of Biopsy	Diagnosis	Fibrosis	Basal cell hyperplasia	Inflammation	Endoscopy
Eso1	50/F	10/20/2010	Schatzki's ring	NApp	0	8	Schatzki's
				2	1	12	
Eso2-lower	78/F	12/9/2010	Lupus/GER	NA			Barrett's, hiatal hernia
Eso3-upper	45/F	12/16/2010	Anemia	NA	NA	NA	Erythema
Eso4-lower	24/M	2/10/2011	GER	NApp	0	6	Reactive epithelial Δ
Eso5	31/F	3/24/2011	SpA/GER	NA			Hiatal hernia
Eso6-lower	34/M	1/11/2012	GER	1	1	22	Reactive epithelial Δ
Eso7	24/F	1/12/2012	GER	NApp	0	7	Reactive epithelial Δ
				NApp	1	8	
dSScEso1*	52/F	3/23/2010	dcSSc	NApp	1	3	GAVE
				NApp	0	0	
dSScEso2-lower	30/F	9/9/2010	dcSSc	1	1	2	Barrett's
dSScEso3	49/F	3/24/2010	dcSSc	NApp	0	8	Hiatal hernia, stricture
				1	1	3	
lSScEso4	53/F	9/8/2010	lcSSc	NApp	1	6	Hiatal hernia, stricture
				NApp	1	10	
lSScEso5	58/F	9/30/2010	lcSSc	NApp	0	1	Hiatal hernia
				NApp	0	3	
lSScEso6	63/M	10/14/2010	lcSSc	NApp	0	0	Candida esophagitis, stricture
				3	2	5	
dSScEso8-lower	50/F	10/20/2010	dcSSc	1	1	19	Reflux esophagitis
lSScEso9-upper	52/F	2/16/2011	lcSSc	1	1	14	Reactive epithelial Δ
dSScEso11-lower	68/F	2/16/2011	dcSSc	2	1	25	Candida esophagitis
dSScEso12	49/F	4/20/2011	dcSSc	NA			GAVE

lSScEso13-unknown	47/F	11/22/2011	lcSSc	NApp	1	7	Reactive epithelial Δ
dSScEso14-lower	46/F	1/9/2012	dcSSc	1	0	4	Reactive epithelial Δ
dSScEso15-unknown	50/F	1/25/2012	dcSSc	1	1	6	Reactive epithelial Δ
dSScEso17	67/F	8/1/2012	dcSSc	NA			
dSScEso18	51/F	8/9/2012	dcSSc	NA			Hiatal hernia
dSScEso19-lower	51/F	9/26/2012	dcSSc	1	2	27	

* indicates subject had baseline and 6mo follow-up biopsies, **subject age at the time of biopsy. GER=gastroesophageal reflux disease, NApp=Not applicable, NA=not available, SpA=spondyloarthropathy, dSSc= diffuse cutaneous systemic sclerosis (SSc), lSSc=limited cutaneous SSc. Split cells per patient indicate upper and lower esophageal biopsies were performed. One cell per patient indicates lower or upper esophageal biopsies were performed as indicated. GAVE=gastric antral vascular ectasia syndrome (watermelon stomach).
